# Synthesis, Characterization, and Biological Effects of Chloro-Cathinones: Toxicity and Potential Neurological Impact

**DOI:** 10.3390/ijms26083540

**Published:** 2025-04-09

**Authors:** Ana Patrícia Gomes, Raquel Ferro, Daniela Pinto, Joana Silva, Celso Alves, Rita Pacheco, Helena Gaspar

**Affiliations:** 1 BioISI—Biosystems and Integrative Sciences Institute, Faculdade de Ciências, Universidade de Lisboa, Campo Grande, 1749-016 Lisboa, Portugal; a43724@alunos.isel.pt (A.P.G.); raquel.andreia.ferro@gmail.com (R.F.); fc51313@alunos.ciencias.ulisboa.pt (D.P.); 2 Centro de Química Estrutural, Institute of Molecular Sciences, Universidade de Lisboa, Campo Grande, 1749-016 Lisboa, Portugal; 3 Departamento de Engenharia Química, Instituto Superior de Engenharia de Lisboa, Av. Conselheiro Emídio Navarro, 1959-007 Lisboa, Portugal; 4 MARE—Marine and Environmental Sciences Centre, ARNET—Aquatic Research Network, ESTM, Polytechnic University of Leiria, 2520-614 Peniche, Portugal; joana.m.silva@ipleiria.pt (J.S.); celso.alves@ipleiria.pt (C.A.)

**Keywords:** NPS, cathinones, NMR, GC-MS, HRMS, SH-SY5Y, acetylcholinesterase

## Abstract

Cathinones, a class of synthetic new psychoactive substances (NPSs), continue to emerge and pose public threats. Government control efforts often lead to the emergence of new isomers, which have adverse repercussions on NPSs identification and risk prediction. This work reports on the synthesis and structural characterization of twenty chloro-cathinones, including different isomers, to create analytical data to facilitate their identification in forensic and clinical contexts. Additionally, the potential of these cathinones to cause neuronal damage was evaluated. In vitro cytotoxicity was assessed using a differentiated human neuroblastoma cell line (SH-SY5Y) as a dopaminergic neuronal model. The tested cathinones showed LC_50_ values from 0.6 to 2.5 mM, with 4-CBC being the most cytotoxic. The most toxic cathinones increase reactive oxygen species levels and/or cause mitochondrial membrane potential depolarization. Furthermore, this study explored, for the first time, the effect of cathinones on the cholinergic system through acetylcholinesterase (AChE) inhibition. All tested cathinones inhibited AChE with IC_50_ values between 0.1 and 2 mM. Molecular docking analysis revealed that the most inhibitory cathinones interacted with the CASs and PASs in AChE’s active gorge. These findings provide valuable insights into the effects of cathinones, highlighting potential health risks and structural features that may influence their toxicity towards the cholinergic system and neuronal damage.

## 1. Introduction

Synthetic cathinones are a class of new psychoactive substances (NPSs) that emerged as an alternative to amphetamines and other controlled psychostimulant drugs, with the intention of mimicking their psychoactive effects and circumventing legislation [1]. These substances are chemically analogous to naturally occurring cathinone, the main psychoactive alkaloid found in the khat plant (*Catha edulis*) [2].

By the end of 2023, the European Monitoring Center for Drugs and Drug Addiction (EMCDDA) (recently substituted by the European Union Drugs Agency—EUDA) was monitoring about 950 NPSs. Of the twenty-six substances reported for the first time that year, three were synthetic cathinones. Despite being the second largest category of NPSs monitored by the European Early Warning System (EWS), large quantities of cathinones, such as 3-MMC, 3-CMC, and 4-CMC, were seized in 2021, making them the most apprehended NPSs. This trend of significant cathinone seizures persisted in 2023 [3].

The consumption of synthetic cathinones can interfere with various mechanisms in the brain. In particular, they affect intercellular communication by influencing neurotransmitter release, receptor binding, and the elimination mechanisms within the synaptic cleft [4].

Similarly to other central nervous system (CNS) psychostimulants, synthetic cathinones disturb pathways associated with the monoamines norepinephrine (NA), serotonin (SER), and dopamine (DA) [5,6,7]. This interference leads to an increase in extracellular monoamine levels in the synaptic cleft [8,9,10], thereby enhancing signaling between neuronal cells. Several studies have already been published, demonstrating that the neurotoxic effects of cathinones on monoamine transporters within neuronal cells are like those of other illicit drugs, such as amphetamines, cocaine, and MDMA, but with different potencies and effects. Moreover, the magnitude and selectivity of these interactions differ depending on the specific type of cathinone [6,11,12,13].

The continuous emergence of cathinones and other NPSs raises significant concern among authorities due to the limited understanding of their effects and the perceived potential harm associated with their consumption, as evidenced by the increased incidence of intoxication [3]. Therefore, there is an urgent need to comprehensively investigate their biological effects and toxicity.

Generally, the consumption of illicit drugs is commonly linked to the dopaminergic system, associated with DA neurotransmitters. This system is part of both the CNS and peripheral nervous system (PNS) and is recognized to be involved in several brain functions, including reward, cognition, and motivation [14]. However, drug dependence has been linked to the complex interaction between the dopaminergic and cholinergic system [15]. The cholinergic system, with acetylcholine (ACh) as its neurotransmitter, has already been implicated in nicotine dependence [16], and some have already reported on the potential role of ACh as a treatment target for addictive stimulants, specifically cocaine and amphetamines [17].

The cholinergic system found in various parts of the nervous system, namely the CNS, PNS, and the neuromuscular junction, is involved in sensory and motor processing, sleep and mood regulation, stress response, attention, arousal, and memory, as well as motivation and reward [15,17]. The enzyme acetylcholinesterase (AChE) plays a crucial role in the cholinergic system, regulating the levels of ACh in the synaptic cleft. The hydrolysis of ACh by AChE is essential for the proper functioning of the CNS, as it is responsible for returning the cholinergic neuron to its resting state after a nerve impulse. Its inhibition can induce an overstimulation of receptors due to increased ACh concentration and interaction time in the synaptic cleft [18]. While reversible AChE inhibitors have shown therapeutic interest for the treatment of neurodegenerative diseases, such as Alzheimer’s and Parkinson’s diseases, irreversible inhibition is associated with toxic effects, potentially causing neurodegeneration and even death [18].

An excessive accumulation of ACh, resulting from a prolonged inhibition of AChE, has been linked to a range of effects, including headache, insomnia, dizziness, confusion, and fatigue. In more severe cases, it can lead to CNS depression, manifesting as slurred speech, convulsions, coma, and respiratory depression [19]. Notably, these symptoms bear resemblance to those observed in individuals experiencing adverse events associated with the consumption of synthetic cathinones [20,21,22], suggesting a potential link between cathinone intake and AChE inhibition-induced neurotoxicity. Despite the extensive research on AChE inhibitors, our understanding of cathinones’ impact on AChE activity within the context of drug abuse remains limited. However, both preclinical and clinical studies have reported the use of AChE inhibitors to treat cognitive impairment associated with drug dependence [23,24,25]. While numerous reports link cathinones to the dopaminergic system, information regarding the influence of synthetic cathinones on the cholinergic system and AChE activity is currently lacking.

The aim of the present study was to investigate the neurotoxic potential of twenty chloro-cathinones (Table 1) and to elucidate their impact on neuronal function. Cytotoxicity was assessed in vitro using the SH-SY5Y cell line, differentiated into a dopaminergic neuronal phenotype. Mephedrone was used as a model drug, as this cathinone is associated with widespread misuse and numerous reported cases of intoxication and death [26,27]. The ability of these cathinones to inhibit the enzyme acetylcholinesterase (AChE) was also evaluated. Docking studies were performed for the most inhibitory cathinones to identify possible binding modes of cathinones with residues in the AChE active site to understand the mechanism of cathinone inhibition. To our knowledge, this is the first time that acetylcholinesterase inhibition has been assessed for cathinones. In addition, biomarkers related to mitochondrial membrane potential (MMP) and oxidative stress were investigated to unravel the potential mechanism pathways associated with cytotoxicity.

The cathinones selected for this study (Table 1) were categorized into eight distinct groups of chlorinated cathinones: chloro-*N*-methylcathinones (CMC) (**1**–**3**), chloro-*N*-ethylcathinones (CEC) (**4**–**6**), chloro-*N*-butylcathinones (CBC) (**7**–**8**), chloro-pyrrolidinylcathinones (Cl-PPP) (**9**–**11**), chloro-*N*,*N*-dimethylcathinones (CDC) (**12**–**14**), chloro-*N*,*N*-diethylcathinones (Cl-DEC) (**15**–**16**), chloro-*N*-isopropylcathinones (CIC) (**17**–**18**), and chloro-*tert*-butylcathinones (Cl-TBC) (**19**–**20**). Each of these cathinones shared a common chemical structure backbone of propiophenones, differing only by variations in the amino group at position 2 and/or the location of the chlorine atom in the aromatic ring (*ortho*, *meta*, *para*).

It should be noted that, for the purposes of this study, the selection of cathinones aimed to include those already reported to the EUDA, namely 2-CMC, 3-CMC, 4-CMC, 3-CEC, 4-CEC, 4-CBC, 4-Cl-PPP, 4-CDC, 4-CIC, and 3-Cl-TBC, commonly known as bupropion, an antidepressant medication also used in aiding smoking cessation [29,30]. We also included some of their isomers, which have not yet been seized but are likely to emerge soon on the illicit drug market. All these cathinones were fully characterized by NMR and GC-MS to provide analytical data enabling their further identification in forensic and medical laboratories.

## 2. Results

### 2.1. Neurotoxic Activity on Differentiated SH-SY5Y Cells

**Figure 1:**
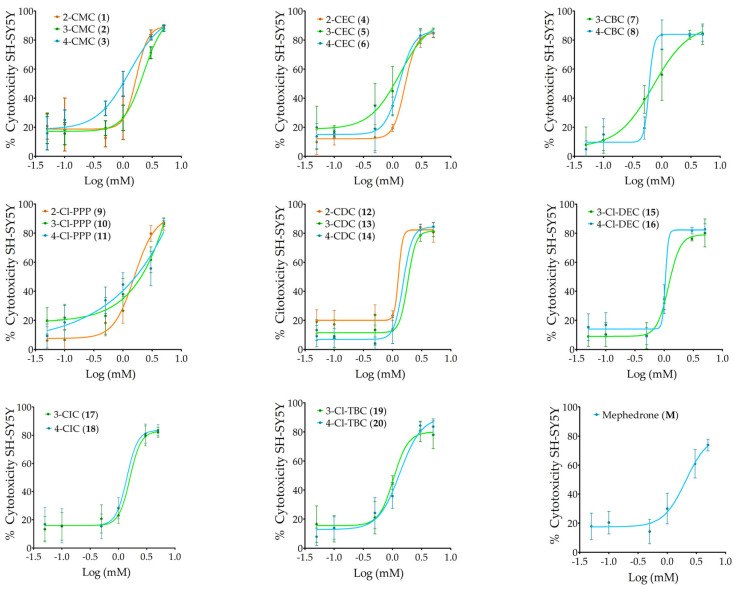
Dose–response curves of the compounds (0.05–5 mM) on the differentiated SH-SY5Y cell line for 24 h. The cytotoxicity percentage was determined by the MTT assay and represented as the mean ± standard deviation (SD) of three independent experiments carried out in triplicate.

The toxicity of twenty chloro-cathinones (Table 1, **1**–**20**) and mephedrone was tested on differentiated SH-SY5Y cells at 0.05–5 mM. The SH-SY5Y human neuroblastoma cell line was induced to differentiate into a dopaminergic neuron-like morphology and used as an in vitro model to evaluate neurotoxicity. The results presented in Figure 1 show the dose–response curves of the cathinones grouped by structural isomers (*ortho*, *meta* and *para*) and mephedrone (**M**).

All cathinones tested showed a concentration-dependent cytotoxic effect, with a maximum response produced over 80% cytotoxicity, with the exception of mephedrone (**M**), which showed a maximum response of 73%. In particular, 4-CBC (**8**), 2-CDC (**12**), and 4-Cl-DEC (**16**) showed steep curves, indicating that a small variation in dose significantly increases their cytotoxicity and, therefore, these cathinones have a higher risk of toxicity compared to others. Parameters such as the concentration of cathinone showing 50% cytotoxicity (LC_50_), Hill slope, and lowest observed adverse effect level (LOAEL) obtained from dose–response curves by adjusting the non-linear sigmoidal Hill regression model are summarized in Table 2.

The most cytotoxic cathinone was 4-CBC (**8**) and its structural isomer 3-CBC (**7**), with LC_50_ values of 0.6 mM and 0.7 mM, respectively, followed by 4-Cl-DEC (**16**) and 3-Cl-TBC (**19**), with LC_50_ values of 1.0 mM. Table 2 also shows that 4-CMC (**3**), 4-CEC (**6**), 3-CBC (**7**), and 3-Cl-PPP (**10**) exhibited significant cytotoxicity at low concentrations, with a LOAEL of 0.1 mM. In addition, a LOAEL of 0.5 mM was observed for 2-CEC (**4**), 3-CEC (**5**), 4-CBC (**8**), 2-Cl-PPP (**9**), 4-Cl-PPP (**11**), 3-Cl-TBC (**19**), and 4-Cl-TBC (**20**), similar to mephedrone (**M**). The remaining cathinones, 2-CMC (**1**), 3-CMC (**2**), 2-CDC (**12**), 3-CDC (**13**), 4-CDC (**14**), 3-Cl-DEC (**15**), 4-Cl-DEC (**16**), 3-CIC (**17**), and 4-CIC (**18**), exhibited significant cytotoxicity with a 1 mM LOAEL.

**Figure 2:**
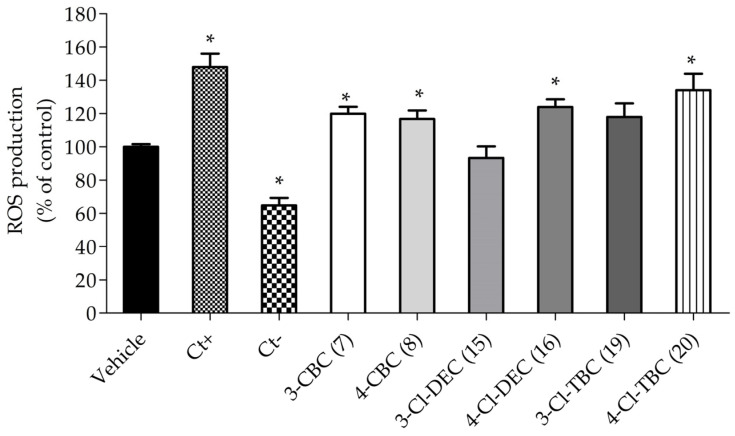
Intracellular reactive oxygen species levels (% of control) after 6 h cell treatment at LC_50_. The effect was estimated using a 2′,7′- dichlorofluorescein diacetate (carboxy-H2DCFDA) probe. Ct+: hydrogen peroxide; Ct-: ascorbic acid. The values in each column represent the mean ± standard error of the mean (SEM) of three independent experiments carried out in triplicate. * *p* < 0.05 indicates a significant difference when compared to vehicle.

**Figure 3:**
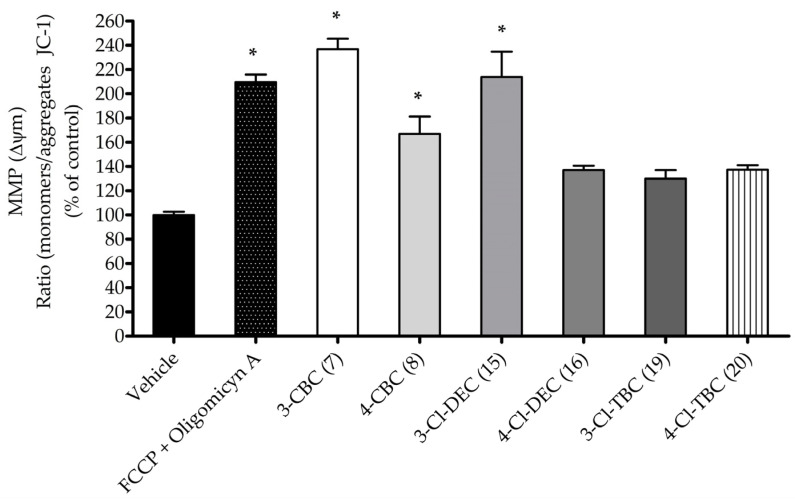
Alterations in mitochondrial membrane potential after 6 h cell treatment at LC_50_. The effect was estimated using JC-1 probe. FCCP (2.5 µM) and oligomycin A (1 mg/mL) conjugate solution was used as positive control. The values in each column represent the mean ± standard error of the mean (SEM) of three independent experiments carried out in triplicate. * *p* < 0.05 indicates a significant difference when compared to vehicle.

In order to understand the mechanisms underlying cathinone neurotoxicity, the most cytotoxic cathinones and their isomers, CBC (**7**–**8**), Cl-DEC (**15**–**16**), and Cl-TBC (**19**–**20**), were evaluated at LC_50_ for their impact on oxidative stress induction and their effects on mitochondrial membrane potential (MMP). This study was performed after 6 h treatment of SH-SY5Y cells to detect early alterations in cellular stress and understand the initial mechanisms of action leading to cell death. The obtained results are shown in Figure 2 and Figure 3.

In Figure 2, it is possible to observe that the exposure of SH-SY5Y cells to cathinones after 6 h treatment significantly increased ROS production by 10–30% compared to the vehicle, with the exception of 3-CI-DEC (**15**). However, when we compared the effect between all cathinones, 4-CI-TBC (**20**) showed an increase in ROS level production of 30%.

To further investigate the neurotoxic effects of cathinones, the alterations of MMP in cells exposed to the LC_50_ concentration of cathinones was evaluated. Mitochondrial membrane potential depolarization is a key indicator of mitochondrial dysfunction which is closely linked to cell death in both apoptosis and necrosis. The results, presented in Figure 3, show a significant depolarization of MMP in cells treated with cathinones, with values ranging from 50% to 240% compared to vehicle. Particularly, 3-CBC (**7**), 4-CBC (**8**), and 3-CI-DEC (**15**) caused a more pronounced MMP depolarization than the other cathinones. The magnitude of this depolarization was comparable to that observed with the positive control, a combination of carbonyl cyanide-4-(trifluoromethoxy)phenylhydrazone (FCCP), and oligomycin A, which are known to disrupt mitochondrial function.

### 2.2. Acetylcholinesterase (AChE) Inhibitory Activity

**Figure 4:**
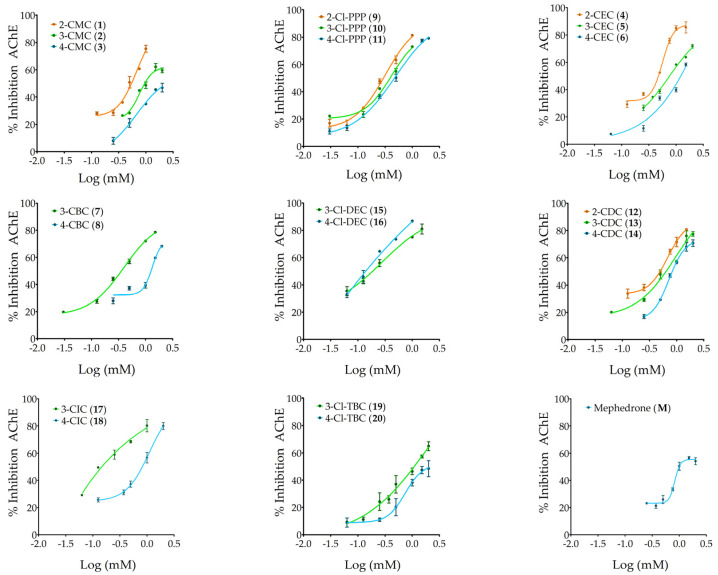
Dose–response curves—percentage of AChE inhibition effects (%) of the 20 cathinones (0.03 to 2 mM) grouped by isomer sets and of mephedrone on AChE activity. The percentage inhibition for each concentration is represented as the mean ± standard deviation (SD) of at least two tests performed.

The ability of twenty-one cathinones to inhibit acetylcholinesterase activity was tested at concentrations ranging from 0.03 to 2 mM. The results in Figure 4 show that all analyzed cathinones inhibited AChE activity in a dose-dependent manner. Among the tested concentrations, the cathinones exhibiting the highest maximum inhibition were 3-Cl-DEC (**15**), 2-CEC (**4**), and 2-Cl-PPP (**9**), reaching 87%, 85%, and 81% inhibition, respectively.

Table 3 summarizes the parameters obtained by fitting the non-linear sigmoidal Hill regression model to dose–response curves, the concentration of cathinone that inhibits 50% the enzyme activity (IC_50_), LOAEL, and the Hill slope. For cathinones 4-CMC (**3**) and 4-Cl-TBC (**20**), at the highest tested concentration of 2 mM, 47% and 48% inhibition in acetylcholinesterase activity was achieved, respectively.

The cathinones showing the highest inhibition of AChE activity, and therefore the lowest IC_50_ values, were 4-Cl-DEC (**16**), followed by 3-Cl-DEC (**15**) and 3-CIC (**17**), with IC_50_ values of 0.1 mM, 0.2 mM, and 0.3 mM, respectively, and with no significant difference between the IC_50_ values of the last two cathinones. Furthermore, the cathinones with the lowest IC_50_ values, hence considered the most inhibitory, were also those exhibiting the lowest LOAEL value of 0.063 mM. Other cathinones, including 3-CBC (**7**), 4-CBC (**8**), and 2-Cl-PPP (**9**), also displayed a low LOAEL of 0.100 mM, while 3-Cl-PPP (**10**) exhibited a LOAEL of 0.125 mM. The majority of cathinones, such as 3-CMC (**2**), 2-CEC (**4**), 4-CDC (**14**), and 4-Cl-TBC (**20**), showed enzyme inhibition beginning at 0.500 mM, with mephedrone (**M**) having the highest LOAEL determined, at 0.750 mM.

Given the Hill slope values obtained, the most inhibitory cathinones 4-Cl-DEC (**16**), 3-Cl-DEC (**15**), and 3-CIC (**17**), with values close to 1, suggest a non-cooperative inhibition mode towards AChE. However, the majority of the cathinones exhibited Hill slope values above 1, indicating positive cooperative inhibition. In this mechanism, the binding of one cathinone molecule facilitates the binding of subsequent molecules, given AChE’s oligomeric nature. Notably, mephedrone (**M**) exhibited the highest Hill slope, followed by 4-CBC (**8**), suggesting the high potency of these cathinones, for which even small variations in concentration cause a substantial increase in AChE inhibition.

### 2.3. Molecular Docking Studies

**Figure 5:**
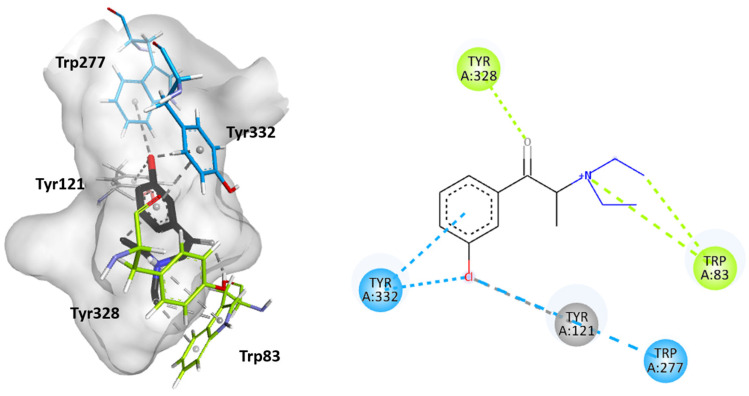
Molecular docking of the binding mode for 3-Cl-DEC (**15**) with AChE (PDB id:4EY7), visualized in Discovery Studio, BIOVIA. Blue indicates PAS residues; green indicates CAS residues; grey indicates mid-gorgeresidues.

**Figure 6:**
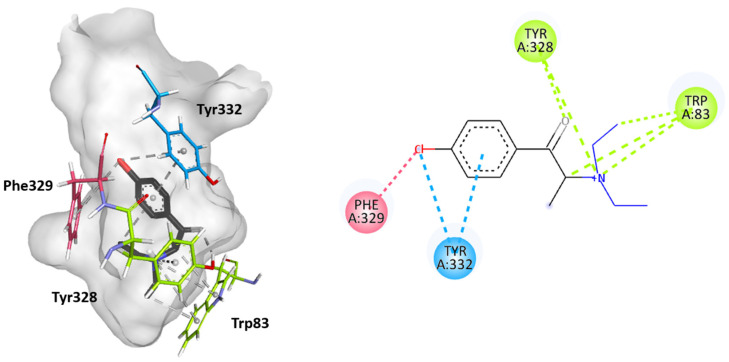
Molecular docking of the binding mode for 4-Cl-DEC (**16**) with AChE (PDB id:4EY7), visualized in Discovery Studio, BIOVIA. Blue indicates PAS residues; green indicates CAS residues; pink indicates acyl binding pocket residues.

**Figure 7:**
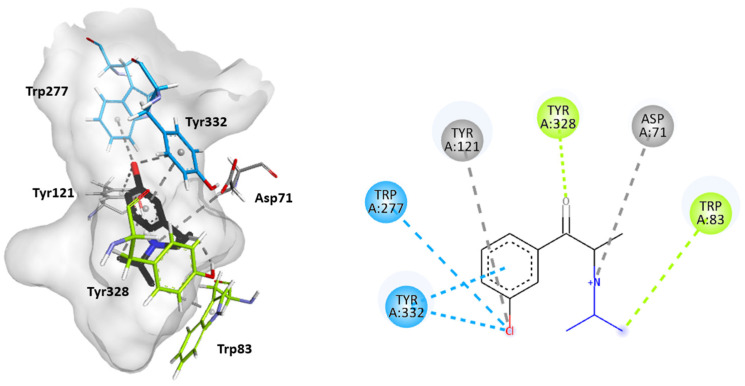
Molecular docking of the binding mode for 3-CIC (**17**) with AChE (PDB id:4EY7), visualized in Discovery Studio, BIOVIA. Blue indicates PAS residues; green indicates CAS residues; gray indicates mid-gorge residues.

**Figure 8:**
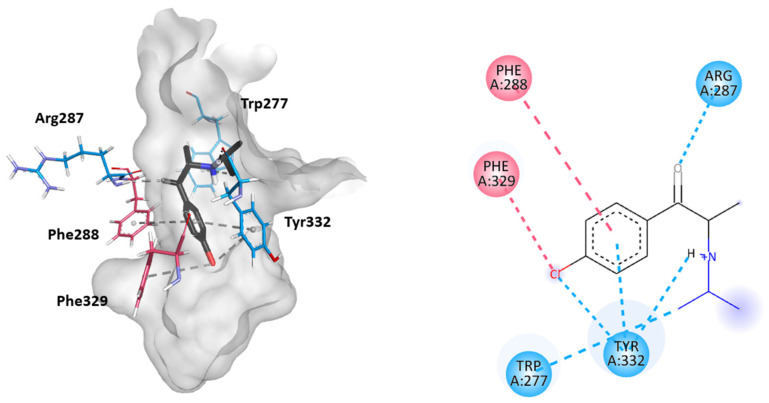
Molecular docking of the binding mode for 4-CIC (**18**) with AChE (PDB id:4EY7), visualized in Discovery Studio, BIOVIA. Blue indicates PAS residues; pink indicates acyl binding pocket residues.

Molecular docking studies were performed to further elucidate the interactions between cathinones and the AChE enzyme active site gorge for the most AChE-inhibitory cathinones, i.e., 3-Cl-DEC (**15**), 4- Cl-DEC (**16**), and 3-CIC (**17**). The same study was performed for 4-CIC (**18**), which is an isomer of 3-CIC but which exhibits lower inhibitory potential. Based on the docking score, the best binding conformation for each cathinone at the AChE active site gorge is shown in Figure 5, Figure 6, Figure 7 and Figure 8. The type of interactions and respective distances are summarized in Tables S10–S13 (see Supplementary Materials).

For AChE, the binding interactions of substrates or inhibitors in the active site are relatively well understood. The AChE active site is characterized as a deep gorge. At the bottom of the gorge, near the acyl-binding pocket and opposite to the catalytic anionic site (CAS), are three amino acids residues form the catalytic triad, Ser200, Glu327, and His440. A peripheral anionic site (PAS) occurs at the entrance of the active site, and several amino-acid residues are displayed along the mid-gorge, forming a narrow-elongated cavity [31,32]. The interaction with substrates like ACh typically begins at the PAS and then progresses to the CAS, and the acetyl group of substrate ACh binds to the aromatic amino acid residues in the acyl-binding pocket prior to ACh hydrolysis. In the case of inhibitors, these are reported to interact with both the PAS and CAS [33].

The docking analysis indicated that the cathinones interact with multiple amino acid residues within the AChE gorge. Interestingly, for the most inhibitory cathinones, both isomers of Cl-DEC (**15**–**16**) and 3-CIC (**17**), it was found that all had their amino moiety oriented towards the CAS and positioned towards the bottom of the gorge, while these cathinones’ aromatic rings interacted with the PAS and mid-gorge residues. Conversely, 4-CIC (**18**), which experimentally showed significantly weaker AChE inhibition, showed different interactions, with its amino moiety positioned towards the entrance of the gorge and mainly interacting with the PAS.

Figure 5 and Figure 6 show that both isomers Cl-DEC (**15**–**16**) within the AChE active site form a hydrogen bond between their carbonyl group and Tyr328 residue located in the CAS region. 4-Cl-DEC (**16**) displays an additional π-cation interaction between its amino moiety and this amino acid residue. Additionally, both isomers (**15**–**16**) display π-sigma and π-cation interactions between their amino moiety and the Trp83 residue also located in the CAS region, with 4-Cl-DEC (16) demonstrating a further π-sigma interaction with this amino acid residue. The cathinones’ (**15**–**16**) aromatic rings stack with Tyr332, located in the PAS region, and their chlorine atoms also establish a π-Cl interaction with this amino acid residue. Additional interactions were seen to involve the chloride atom, either with the Trp277 residue located in the PAS region or with Tyr121 positioned within the mid-gorge for 3-Cl-DEC (**15**); for 4-Cl-DEC (**16**), a π-Cl interaction occurred with the aromatic residue Phe329 located in the acyl binding pocket.

Figure 7 reveals that for 3-CIC (**17**), which also exhibits high AChE-inhibitory activity, similar interactions occur in the CAS and PAS regions, as observed for the Cl-DEC isomers (**15**–**16**). Specifically, the amino acid residues Tyr328 and Trp83 located in the CAS region interacted by hydrogen bonds and π-sigma interactions, respectively, with the carbonyl group and the amino moiety of the cathinone. Further electrostatic interaction occurred between the amino moiety of 3-CIC (**17**) and Asp71 located in the mid-gorge. Also, the aromatic ring of this cathinone interacts with the amino acid residues Tyr332 and Trp277, located in the PAS region, either by π-π stacking or by π-Cl interactions. The chloride atom in the aromatic ring of the cathinone also showed interactions with Tyr121 when the ring was positioned within the mid-gorge.

As seen in Figure 8, in the case of 4-CIC (**18**), a weaker AChE inhibitor, interactions primarily occur within the PAS region. The carbonyl group of the cathinone interacts with the Arg287 residue by hydrogen bonding. Furthermore, in this region, the Trp277 and Tyr332 residues interact with the amino moiety of the cathinone. The latter also exhibits interactions with the cathinone aromatic ring by π-π interaction and with the chloride atom through π-Cl interaction. Additionally, the aromatic ring of 4-CIC (**18**) is stacked with the aromatic amino acid residue Phe288 located in the acyl binding pocket. Moreover, the Phe329 residue from the acyl binding pocket also displays a π-interaction with the chlorine atom in the aromatic ring.

## 3. Discussion

Cathinones, among other synthetic NPSs, are continually emerging and consistently causing public threats. Once a government regulates a specific cathinone, the likelihood of a new replacement, such as its isomers, emerging increases. The identification and effective control of NPSs in forensic and medical laboratories are challenged by the dynamics of this illicit market. In addition, the unforeseen effects of the chemical structure modifications of cathinones pose significant risks to consumer health. As such, this study synthesized and characterized cathinones that have already been reported to the EUDA [2-CMC (**1**), 3-CMC (**2**), 4-CMC (**3**), 3-CEC (**5**), 4-CEC (**6**), 4-CBC (**8**), 4-Cl-PPP (**11**), 4-CDC (**14**), 4-CIC (**18**), and 3-Cl-TBC (**19**)], along with their potential isomeric forms [2-CEC (**4**), 3-CBC (**7**), 2-Cl-PPP (**9**), 3-Cl-PPP (**10**), 2-CDC (**12**), 3-CDC (**13**), 3-Cl-DEC (**15**), 4-Cl-DEC (**16**), 3-CIC (**17**), and 4-Cl-TBC (**20**)], as they may arise in the illicit drug market.

The objective of this work was also to assess the potential toxicity of these cathinones in a neuronal cellular model and to evaluate their effects on the inhibition of AChE, a critical enzyme in the cholinergic system. Additionally, we aimed to investigate whether structural modifications of the cathinones are associated with the observed effects.

The results demonstrated that most of the isomers of the tested cathinones, not yet reported to the EUDA, exhibit cytotoxicity against the dopaminergic model of differentiated SH-SY5Y cells. Among them, the CBC (**7**–**8**), Cl-DEC (**15**–**16**) and Cl-TBC (**19**-**20**) isomers were seen to be the most cytotoxic, exhibiting the lowest LC_50_ values.

When comparing the isomers regarding the position of the chloride atom in the cathinone aromatic ring and their LC_50_ values (Table 2), generally, it was seen that the *meta*- isomers exhibit lower cytotoxicity compared to other ring-positional isomers. This trend was particularly noticeable for 3-Cl-PPP (**10**), with an LC_50_ of 2.5 mM, compared to 2-Cl-PPP (**9**) and 4-Cl-PPP (**11**), which had LC_50_ values of 1.6 mM and 1.8 mM, respectively.

Furthermore, the cytotoxicity results in differentiated SH-SY5Y cells suggest that effect is linked to the nature of the substituted amino group. For example, an increased cytotoxicity is induced by the elongation of the aliphatic linear chains in *N*-alkyl and *N*,*N*-dialkyl cathinones; it is noteworthy that cathinones with *N*-alkyl chains exhibit higher cytotoxicity when compared to those with *N*,*N*-dialkyl chains. This is particularly evident when comparing the LC_50_ values of CBC (**7**–**8**), 0.7 and 0.6 mM, with those of Cl-DEC (**15**–**16**), 1.2 and 1.0 mM. Similarly, CEC (**5**, **6**) demonstrate higher cytotoxicity (LC_50_ of 1.3 mM) than CDC (**13**, **14**) (LC_50_ of 1.8 and 1.5 mM). Regarding the pyrrolidinyl cathinones with a cyclic amino group, Cl-PPP (**9**–**11**), it was found that they exhibit decreased cytotoxicity compared to cathinones with linear *N*,*N*-dialkyl chains, such as CDC (**12**–**14**) or Cl-DEC (**15**–**16**).

**Figure 9:**
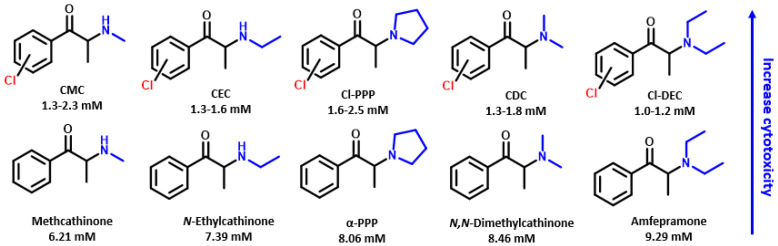
Influence of the chlorine atom in the aromatic ring on the cytotoxicity of different cathinone analogs: a comparison of our results with those resported in literature [34].

In a previous investigation, the toxicity of other cathinones indicated a correlation between shorter alkyl substituents on the amino group and increased toxicity, such as between amfepramone and *N*,*N*-dimethylcathinone, as well as between *N*-ethylcathinone and methcathinone [34]. Our results showed a contradictory trend, specifically for cathinones with shorter amino group, such as CMC (**1**–**3**), exhibiting lower cytotoxic than CEC (**4**–**6**) and consequently being less cytotoxic than CBC (**7**–**8**), while CDC (**12**–**14**) demonstrated lower cytotoxicity than Cl-DEC (**15**–**16**), within the same set of *ortho*, *meta*, and *para* isomers, probably due to the chlorine atom present in the aromatic ring. As mephedrone showed a comparable toxicity in the previous study [34], a comparison of the LC_50_ values obtained for CMC (**1**–**3**), CEC (**4**–**6**), Cl-PPP (**9**–**11**), CDC (**12**–**14**), and Cl-DEC (**15**–**16**) with those of structurally analogous non-chlorinated cathinones (Figure 9) suggests that the inclusion of a chlorine atom in the aromatic ring significantly increases cytotoxicity, as our study yielded lower LC_50_. This correlation is further supported by comparisons of the LC_50_ values of chloro-cathinone CMC (**1**–**3**) with mephedrone (**M**).

Moreover, a recent study by Chojnacki et al. [35] on the pharmacological effects of 4-chloro-cathinones in rats compared the effect of 4-CMC (**3**), 4-CEC (**6**), and 4-Cl-PPP (**11**) with that of mephedrone, suggesting that they may exhibit abuse potential and adverse effects similar to those of mephedrone, though their toxic effects are still unknown.

Among the cathinones studied, CBC (**7**–**8**), Cl-DEC (**15**–**16**), and Cl-TBC (**19**–**20**) were seen to be the most cytotoxic. The potential mechanisms of cytotoxicity were investigated, and it was found that these cathinones induce oxidative stress in dopaminergic differentiated SH-SY5Y cells, as evidenced by an increase in intercellular ROS levels after 6 h of exposure. However, 3-Cl-DEC (**15**) was an exception, suggesting that oxidative stress is not involved in the neurotoxicity mediated by this cathinone. Our findings demonstrated that 3-Cl-DEC (**15**), as well as 3-CBC (**7**) and 4-CBC (**8**), significantly disrupt mitochondrial function, potentially contributing to their neurotoxic effects. Since mitochondrial dysfunction can lead to both apoptotic and necrotic cell death, these results provide a basis for future studies to further investigate the toxicity mechanisms of these cathinones. Future research should focus on assessing morphological changes and key apoptotic biomarkers, such as caspase-3 activity and DNA fragmentation or condensation assays, to gain deeper insights into these cathinones’ neurotoxic potential.

Overall, these mechanisms of cathinone neurotoxicity were also reported for several other cathinones [36,37,38,39]. Multiple studies have investigated the neurotoxic effects of various cathinones. Research on mephedrone and methylone revealed reduced mitochondrial respiration in undifferentiated SH-SY5Y cells [37], while other studies found that methylone and MDPV induce mitochondrial dysfunction and oxidative stress with ROS and RNS production [40]. Cathinones like butanone, pentanone, and 3,4-methylenedioxypyrovalerone were shown to cause dose-dependent neurotoxicity characterized by ROS production and decreased mitochondrial bioenergetics [38]. Additionally, 13 cathinones (amfepramone, buphedrone, mephedrone, methcathinone, *N*,*N*-dimethylcathinone, pentedrone, α-PBP, α-PPP, α-PVP, 3,4-DMMC, flephedrone, methedrone, and *N*-ethylcathinone) were found to induce mitochondrial and lysosomal dysfunction [34]. Studies also demonstrated that cathinones such as 4-F-PV8, 4-F-PV9, 4-F-α-PVP, 4-MeO-PV8, 4-MeO-PV9, 4-MeO-α-PVP, PV8, PV9, and α-PVP induce mitochondrial dysfunction and significant cell membrane damage [41]. These and our findings highlight the diverse neurotoxic effects of cathinones, including mitochondrial dysfunction and oxidative stress.

This study aimed to demonstrate an additional finding regarding the effects of cathinones on the neurological system. Specifically, it was demonstrated that cathinones may affect the cholinergic system by acting as inhibitors of AChE. All the tested cathinones demonstrated AChE inhibitory capacity, with IC_50_ values ranging from 0.1 to 2 mM. Among them, Cl-DEC (**15**–**16**) and 3-CIC (**17**) displayed the highest inhibitory activity. The experimental results suggest that the amino group moiety of the cathinone may influence their inhibitory potential. Specifically, longer aliphatic chains were associated with greater inhibition, namely in 4-CBC (**7**), with IC_50_ values of 0.4 mM, in comparison to 1.2 mM for 4-CEC (**6**) and an IC_50_ value above 2 mM for 4-CMC (**3**). This trend was further supported by docking studies for Cl-DEC (**15**, **16**) and 3-CIC (**17**), which revealed that the most potent cathinones interact significantly between their amino moiety with amino acid residues Trp83 and Tyr328 located in the CAS of the AChE active site gorge.

The experimental data also showed that less branched *N*-alkylated cathinones exhibited a stronger inhibition of AChE, since CIC (**17**–**18**) exhibited higher inhibition (IC_50_ of 0.2 and 1.1 mM) compared to Cl-TBC (**19**–**20**) (IC_50_ of 1.0 and over 2 mM). Additionally, higher carbon atom counts in *N*,*N*-dialkyl cathinones appear to intensify enzyme inhibitory effects, evident for Cl-DEC (**15**–**16**), with IC_50_ of 0.3 and 0.1 mM, when compared to CDC (**13**–**14**), with IC_50_ of 0.9 and 0.7 mM. Comparing 4-CMC (**3**) and mephedrone (**M**), substituting a methyl group with a chlorine atom in the aromatic ring appears to diminish enzyme inhibition activity.

Docking results also indicated that the most potent cathinones show key interactions between their aromatic chlorophenyl moiety and amino acid residues within the PAS. Although, in these predictions, none of the cathinones directly interact with the catalytic triad, the simultaneous interactions with both the CAS and PAS may explain the high inhibitory effects. In contrast, for 4-CIC (**18**), the positioning of the chloride ion seems to obstruct the cathinone from properly reaching the CAS, leading to a reduced AChE inhibition compared to other cathinones. These findings parallel conclusions drawn for the drug donepezil, a well-known inhibitor of AChE activity, as it has been reported to interact with both the CAS and PAS [42,43,44].

Currently, there is limited information available regarding the effect of cathinones on AChE activity, and in the broader context of drugs of abuse, our understanding of their impact on the cholinergic system remains scarce. Javed et al. [45] conducted a study comparing groups of healthy individuals with consumers of heroin, hashish, and other drugs and observed higher AChE activity in the brain of drug-consuming individuals. Similar findings were observed in vivo with *Sprague Dawley* rats exposed to *Cannabis sativa* resin [46]. Research on cocaine and amphetamines has primarily focused on their impact on acetylcholine release, revealing an increase in the release of this neurotransmitter after the consumption of these illicit substances. However, there is limited information available regarding their specific effect on AChE [47].

To the best of our knowledge, this study represents the first investigation into the inhibitory effects of cathinones on AChE activity and provides insights into the underlying mechanism of inhibition.

When comparing the results of the LC_50_ value for cytotoxicity against the dopaminergic model of differentiated SH-SY5Y cells with the IC_50_ AChE inhibition for each cathinone, it was noticed that the IC_50_ values for AChE inhibition are overall lower than the LC_50_ value for neuronal cell cytotoxicity. This highlights an important conclusion regarding cathinone consumption, namely that cathinones at lower concentration may lead to consequences at the level of the cholinergic system prior to inducing neuronal cell damage.

Nevertheless, as cathinones induce cytotoxicity through mechanisms such as oxidative stress and disruption of mitochondrial membrane potential, resulting in ATP depletion and calcium homeostasis dysregulation, these effects can further exacerbate cholinergic system dysfunction, leading to neurodegeneration and neuronal loss. The toxicity of cathinones to the cholinergic system may encompass a broad spectrum of effects on neurons, muscle cells, and other tissues with cholinergic receptors. These toxic effects may include peripheral manifestations, such as muscle weakness and paralysis, as well as cholinergic stimulation leading to symptoms like confusion, agitation, seizures, and coma.

## 4. Materials and Methods

All reagents and solvents used to synthesize cathinones were acquired commercially (analytical-grade) and did not require further purification. These reagents and solvents were obtained from Sigma^®^Aldrich (St. Louis, MO, USA), Riedel-de Haën (Seelze, Germany), and VWR (Radnor, PA, USA).

Dulbecco’s Modified Eagle Medium (DMEM) and fetal bovine serum (FBS) were obtained from Biowest (Nuaillé, France), Roswell Park Memorial Institute (RPMI-1640); trypsin and glutamine were purchased from Biowhittaker^®^ Lonza (Basel, Switzerland). Phosphate-buffered saline (PBS) was obtained from Corning (Corning, NY, USA). Antibiotic–antimycotic solution 100× (10,000 U/mL penicillin, 10 mg/mL streptomycin, and 25 μg amphotericin B/mL), acetylcholinesterase (AChE) (149 U/mg solid, 241 U/mg protein), acetylthiocholine iodide (AChI), 12-O-tetradecanoylphorbol-13-acetate (TPA), and retinoid acid (RA) were obtained from Sigma^®^Aldrich (St. Louis, MO, USA). 5-5′-Dithiobis (2-nitrobenzoic acid) (DTNB) was purchased from Alfa-Aesar (Ward Hill, MA, USA). (4,5-dimetylthiazol-1-yl)-2,5-diphenyltetrazolium (MTT) was obtained from VWR (Radnor, PA, USA). Magnesium chloride-6-hydrate was obtained from Riedel-de Haën (Seelze, Germany). Sodium chloride was obtained from Panreac (Glenview, IL, USA).

### 4.1. Synthesis and Characterization of Cathinones

The synthesis of cathinones used in this work was carried out under the protocol of the Faculty of Sciences of the University of Lisbon and the Forensic Science Laboratory of the Portuguese Judicial Police. Cathinones (**1**–**21**) were synthesized according to our previously reported works [48,49,50] in a three-step pathway: the acid-catalyzed bromination of the proper ketone (2-chloropropiophenone, 3-chloropropiophenone, 4-chloropropiophenone, or 4-methylpropiophenone) and a subsequent nucleophilic substitution reaction of the resulting α-bromoketone, without any purification, with the appropriate amine, to give the target cathinone as free base, followed by acid precipitation to obtain its hydrochloride salt.

For the synthesis of each α-bromoketone, the desired ketone was dissolved in dichloromethane and cooled in an ice bath. A drop of hydrobromic acid (48% aqueous solution) and bromine were added, and the mixture was stirred until the bromine color disappeared. Bromine was then added dropwise in a 1:1 molar ratio to the ketone while stirring. The reaction was stirred at room temperature and protected from light for 2–4 h until complete. Finally, the mixture was concentrated under vacuum to yield the α-bromoketone.

The preparation of cathinones as free bases via nucleophilic substitution involved two different procedures. Briefly, to obtain CMC (**1**, **2**, **3**), CEC (**4**, **5**, **6**), CDC (**12**, **13**, **14**), and mephedrone (**M**), a cooled 2 M solution of the proper amine (methylamine, ethylamine, or dimethylamine) in tetrahydrofuran (THF) was added dropwise to the respective α-bromoketone dissolved in dry THF and maintained in an ice bath during the addition (4 mmol of amine to 1 mmol of bromoketone). A similar procedure was used to prepare CBC (**7**, **8**), Cl-PPP (**9**, **10**, **11**), Cl-DEC (**15**, **16**), CIC (**17**, **18**), and Cl-TBC (**19**, **20**), but with a cooled 50% (*v/v*) solution of the appropriate amine (buthylamine, pyrrolidine, diethylamine, isopropylamine, and *tert*-buthylamine) in dry THF and with a different addition ratio (2 mmol of amine to 1 mmol of bromoketone). In both procedures, after the complete addition of the amine solutions, the reaction mixture was kept at room temperature for all cathinones, except for 4-CIC (**18**), 3-Cl-TBC (**19**), and 4-Cl-TBC (**20**), for which the reaction was heated to around 60–65 °C. The remaining experimental procedure was carried out according to the literature [48,50].

All synthesized cathinone hydrochloride salts were analyzed through nuclear magnetic resonance spectroscopy (NMR), gas chromatography coupled to mass spectrometry (GC-MS), and high-resolution mass spectrometry (HRMS).

Characterization by NMR was carried out by 1D/2D NMR spectra acquired on a Bruker Avance 400 spectrometer in DMSO-*d_6_* operating at 400.13 MHz (for ^1^H) and 100.61 MHz (for ^13^C). Chemical shifts (δ) were expressed in ppm, coupling constants (*J*) were expressed in Hz, and the residual signal values of the deuterated solvent DMSO-*d_6_* (δ_H_ 2.50 and δ_C_ 39.52) were used as a reference. To assign all proton and carbon signals (1D (^1^H, ^13^C APT) and 2D (COSY, HMBC, and HSQC)), NMR experiments were performed. The NMR data obtained in DMSO-*d_6_* for 3-CMC [50], 4-CMC [50,51,52], 3-CIC [50], 4-CIC [50], 3-Cl-TBC [50,53,54], 3-CEC [55,56], 4-CEC [57], 4-CBC [58], 4-CDC [59], and mephedrone [49] were in accordance with previously reported data. Nonetheless, the full ^1^H and ^13^C NMR assignments in DMSO-*d_6_* for 2-CMC, 2-CEC, 2-Cl-PPP, 3-Cl-PPP, 4-Cl-PPP, 2-CDC, 3-CDC, 3-CBC, 3-Cl-DEC, 4-Cl-DEC, and 4-Cl-TBC are reported here for the first time. However, some of these cathinones already have NMR data in the literature, either incomplete or in another deuterated solvent: 2-CEC (D_2_O) [60], 3-Cl-PPP (MeOD) [61], 4-Cl-PPP (MeOD, CDCl_3_) [62,63], 3-CDC (MeOD) [61], 3-Cl-DEC (MeOD) [61], and 4-Cl-TBC [61]. The NMR characterization of cathinones (**1**–**20**) is provided in Tables S1–S8, available in the Supplementary Materials.

Cathinones were analyzed by GC-MS using a Shimadzu GC-2010 gas chromatograph coupled to a Shimadzu GCMS-QP2010 Plus spectrometer equipped with a Shimadzu AOC-20i automatic injector from Shimadzu (Nakagyo-Ku-Kyoto, Japan) and a Sapiens 5MS column (30 m × 0.25 mm × 0.25 µm) from Teknokroma Analítica SA (Sant Cugat del Vallès, Spain) using helium as carrier gas with at flow rate of 0.93 mL/min. Methanolic solutions of cathinones were prepared at a concentration of 1 mg/mL and 1 µL of each sample was injected in split mode (1:99). The temperature of the injector was 280 °C, and that of the detector was 250 °C. The GC oven was programmed at 80 °C (1 min) and raised to 140 °C at a gradient of 10 °C/min, a gradient of 5 °C/min to 220 °C, and 40 °C/min to 300 °C (5 min). Mass spectra were acquired by electroionization (EI) at a fixed energy of 70 eV., with acquisition starting at 5 min in full scan mode for a range from 10 to 800 *m*/*z*. Shimadzu’s GCMSSolution 2.32 Post Run Analysis software was used to process the data.

The EI-MS of all cathinones (Figures S1–S8 Supplementary Materials) showed the fragmentation pattern characteristic of cathinones. The molecular ion was absent and the two diagnostic peaks resulting from Cα-Cβ cleavage were present: acylium ion (*m/z* 139/141), and iminium ion. In the isomers of CMC, CEC, CBC, PPP, CDC, and Cl-DEC, the iminium ion, *m/z* 58, 72, 100, 98, 72, and 100, respectively, corresponded to the base peak, as expected. However, in the isomers of Cl-TBC and CIC, the iminium ion (*m/z* 100) underwent secondary fragmentation, yielding an ion of *m/z* 44, which is the base peak in the mass spectrum of these cathinones due to its instability caused by the presence of a tert-butyl or isopropyl group, respectively. Secondary fragmentation of the iminium ion is also observed in the other cathinones, but the fragments are of lower intensity. The acylium ion also loses carbon monoxide to give the chlorophenyl ion (*m/z* 111/113), and this ion can also lose hydrochloric acid to give a fragment ion of *m/z* 75.

The HRMS analysis of target cathinones (**1**–**20**) were attained on Bruker Impact II instrument (Bruker, Bremen, Germany), a hybrid quadrupole time-of-flight (QTOF) mass spectrometer equipped with an electrospray ion source (ESI) by direct flow injection (at 200 μL/h) of cathinone solutions (10 μg/mL) prepared by diluting 1/100 aqueous solutions of 1 mg/mL with methanol. Data were acquired using Data Analysis 4.4 software. Analyses were performed in the positive mode with a range of *m*/*z* ratios from 100 to 1000, with a quadruple ion energy of 5.0 eV, while the collision energy was 10.0 eV. The dry gas flow was 4.0 L/min at 200 °C, and the capillary voltage was 4500 V. Calibration was performed with the following solution: 250 mL water, 250 mL isopropanol, 750 μL acetic acid, 250 μL formic acid, and 0.5 mL 1 mol/L NaOH solution. The HRMS data of cathinones (**1**–**20**) are presented in Table S9 in the Supplementary Materials. The accurate mass of the protonated molecule of each cathinones was obtained with an error of less than 3 ppm, confirming the molecular formula for all synthesized compounds.

### 4.2. Neurotoxic Activity in Human Neuroblastoma (SH-SY5Y) Cells

#### 4.2.1. Cell Culture and Maintenance

The SH–SY5Y cell line (DSMZ code: ACC 209) was supplied by the German Collection of Microorganisms and the Cell Culture GmbH (DSMZ) biobank and was cultured according to the supplier’s information. Cells were grown in DMEM:F12 (Dulbecco’s Modified Eagle’s Medium/F12), supplemented with 10% fetal bovine serum (FBS) and 1% antibiotic–antimycotic in a humidified atmosphere with 5% CO_2_ at 37 °C. Subculture was performed according to biobank instructions when the cultures reached 80–85% of confluence.

#### 4.2.2. Differentiation of Human SH-SY5Y Neuronal Cells

**Figure 10:**
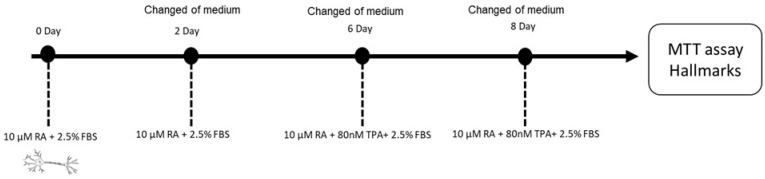
Experimental design of the preparation of differentiated SH-SY5Y cells.

SH-SY5Y cells were trypsinized (trypsin/EDTA; 0.25%) and the cell suspension was seeded in 96-well plates at a density of 2 × 10^4^ cells/well and incubated until reaching 80-85% confluence. Cells were differentiated to obtain a dopaminergic neuronal phenotype according to Ferreira et al. [64] with slight modifications (Figure 10). To ensure reproducibility and optimal neuronal differentiation, cells were used between passages (15–25). Briefly, cells were seeded in complete DMEM:F12 medium containing 10 µM retinoic acid (RA) and 2.5% fetal bovine serum (FBS) and maintained for 4 days, with the medium changed on day 2. After this time, the medium was replaced by DMEM:F12 supplemented with 10 µM RA, 80 nM 12-O-tetradecanoylphorbol-13-acetate (TPA), and 2.5% FBS, and the cells were maintained for an additional 4 days (with the medium changed at day 6). The cytotoxicity assay was performed on day 8.

#### 4.2.3. Cell Viability on Differentiated SH-SY5Y Cells

The SH-SY5Y cells’ viability was evaluated after exposure for 24 h to 5 mM compounds. The 3-(4,5-dimethylthiazol-2-yl) − 2,5-diphenyltetrazolium bromide (MTT) colorimetric assay was performed following the methodology described by Joana Silva et al. [65]. The experimental dose–response results were fitted to the Hill’s sigmoidal non-linear regression model. For each cathinone, the LC_50_ values (cathinone concentration showing 50% lethality) and their confidence interval at the 95% confidence level ([CI]) were determined. The Hill slope, also known as the Hill coefficient, which describes the steepness of a dose–response curve, and the minimum observable adverse effect concentration (LOAEL) were also determined for all cathinones.

#### 4.2.4. 2′,7′-Dichlorodihydrofluorescein Diacetate (H2-DCFDA) Levels

Reactive oxygen species (ROS) production was determined by evaluating ROS levels after exposure to cathinones at concentrations corresponding to their LC_50_ for 6 h. SH-SY5Y cells were washed with 1× PBS and incubated with 100 µL of 2′,7′-dichlorodihydrofluorescein diacetate (H2-DCFDA) (20 µM) in medium without FBS, for 60 min at 37 °C in the dark. Subsequently, the cells were washed with 1× PBS (1×), and the cells were maintained for 30 min at room temperature in 100 µL of PBS. After this time, ROS levels were determined by measuring fluorescence (excitation wavelength (λ): 495 nm; emission λ: 527 nm). Ascorbic acid (A.A) (100 µg/mL) served as the negative control and, hydrogen peroxide (H_2_O_2_) (200 µM) was used as the positive control. Results were expressed as a percentage relative to the control. All data were derived from at least three independent experiments (*n* = 3) and presented as mean ± standard error of the mean (SEM).

#### 4.2.5. Mitochondrial Membrane Potential (MMP)

Mitochondrial membrane potential (MMP) was estimated by using the JC-1 fluorescent probe (Molecular Probes, Eugene, OR, USA) as described in Silva et al., 2022 [65]. The formation of JC-1 aggregates (excitation λ: 490 nm; emission λ: 590 nm) and monomers (excitation λ: 490 nm; emission λ: 530 nm) was measured for 30 min using a plate reader (Bio-Tek Synergy plate reader, Bedfordshire, UK). Changes in MMP were calculated as the ratio between JC-1 monomers and aggregates and expressed as a percentage of the control. An FCCP (2.5 µM) and oligomycin A (1 mg/mL) conjugate solution was used as the positive control. All data were obtained from at least three independent experiments (*n* = 3) and were presented as mean ± standard error of the mean (SEM).

#### 4.2.6. Acetylcholinesterase (AChE) Inhibitory Activity

Acetylcholinesterase (AChE, EC 3.1.1.7) enzymatic activity was assessed using a modified version of Ellman’s colorimetric method, as described by Falé et al. [66]. In this assay, a mixture of 325 μL of 50 mM Tris-HCl buffer (pH 8), 100 μL of a cathinone solution, and 25 μL of AChE (0.1 U/mL in 50 mM Tris–HCl buffer pH 8) were incubated. After a 15 min incubation period, 75 μL of AChI (0.33 mg/mL) was added to 475 μL of DTNB (dithiobis (2-nitrobenzoic acid)) (1.2 mg/mL in Tris–HCl buffer (pH 8) containing 0.1 M NaCl and 0.02 M MgCl_2_). A control reaction, representing 100% activity, was performed using the same volume of water instead of the cathinone solution. The initial velocity of the enzymatic reaction was measured at 405 nm for 5 min, with all assays carried out in duplicate. The percentage of AChE activity inhibition for different concentrations of cathinones [0.03–2 mM] was determined as the ratio of the initial velocity in the cathinone presence to the initial velocity of the control reaction. Experimental results were fitted to Hill’s sigmoidal dose–response non-linear regression model to determine IC_50_ values (cathinone concentration showing 50% inhibition), along with their confidence interval at the 95% confidence level ([CI]). The Hill slope coefficients that provide the steepest value of the slope of the dose–response curve and the minimum observable adverse effect concentration (LOAEL) for all cathinones were also determined.

### 4.3. Statistical Analysis

All data analyses were performed using the software GraphPad v8.0.2 (GraphPad Software, Inc., La Jolla, CA, USA). Statistical significance was defined at the level of 0.05 (*p* < 0.05). All data were checked for normality and homoscedasticity. Non-normally distributed data were compared using the Kruskal–Wallis non-parametric test. One-way analysis of variance (ANOVA) with Dunnett′s multiple comparisons of group means was employed to determine significant differences compared to the control treatment. Post hoc analyses, when applicable, were conducted using Tukey’s test to identify specific group differences.

### 4.4. Molecular Docking Studies

Molecular docking investigations were conducted using AutoDock Vina software (version 1.2.0), CCSB, according to a previously described methodology [67]. The acetylcholinesterase enzyme (PBD ID: 4EY7) was pre-processed for docking simulations using DockPrep in Chimera 1.16 software, while the structures of the cathinones were generated using ChemDraw Ultra 12.0 software. Five models were generated for each cathinone, and the highest-scoring model was chosen for analysis using the Discovery Studio software 2021 (BIOVIA, San Diego, CA, USA).

## 5. Conclusions

This work provides a foundation for future comparisons or discussions, providing relevant data both for the structural identification of cathinones and their impact in neuronal function. Their potential to induce neuronal cell damage and, at a lower concentration, affect the cholinergic system—thereby potentially interfering with neurotransmission and eliciting a broad spectrum of effects in neurons, muscle cells, and other tissues with cholinergic receptors—underscores the urgency for further deepened research into their neurotoxic effects and potential long-term consequences.

It is noteworthy that some of the tested cathinones have already been reported by the EUDA, and others, exhibiting higher toxicity and AChE inhibition than mephedrone and other controlled drugs, such as 4-CMC, may soon emerge on the illicit drug market.

Public awareness of these risks is crucial in mitigating the potential harm posed by cathinones and controlling their illicit market proliferation.

## Figures and Tables

**Table 1:** Structure of synthetic cathinones and date of first seizure reported to EUDA [28].

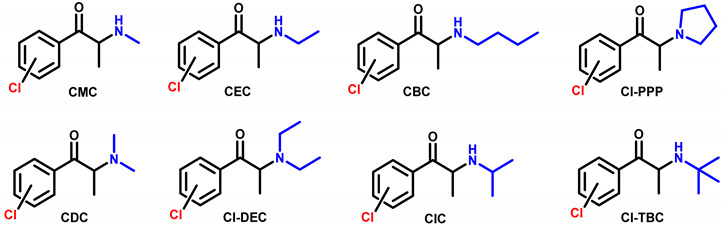					
Cathinone	(Common Name, IUPAC Name)	Structure		Isomer	EUDA Date
		Type	Cl		
2-CMC (**1**)	**2**′-**c**hloro-*N*-**m**ethyl**c**athinone (*R*,*S*)-1-(2-chlorophenyl)-2-(methylamino)propan-1-one	CMC	2′	*ortho*	2024
3-CMC (**2**)	**3**′-**c**hloro-*N*-**m**ethyl**c**athinone (*R*,*S*)-1-(3-chlorophenyl)-2-(methylamino)propan-1-one	CMC	*3′*	*meta*	2014
4-CMC (**3**)	**4**′-**c**hloro-*N*-**m**ethyl**c**athinone (*R*,*S*)-1-(4-chlorophenyl)-2-(methylamino)propan-1-one	CMC	*4′*	*para*	2014
2-CEC (**4**)	**2**′-**c**hloro-*N*-**e**thyl**c**athinone (*R*,*S*)-1-(2-chlorophenyl)-2-(ethylamino)propan-1-one	CEC	*2′*	*ortho*	**-**
3-CEC (**5**)	**3**′-**c**hloro-*N*-**e**thyl**c**athinone (*R*,*S*)-1-(3-chlorophenyl)-2-(ethylamino)propan-1-one	CEC	*3′*	*meta*	2016
4-CEC (**6**)	**4**′-**c**hloro-*N*-**e**thyl**c**athinone (*R*,*S*)-1-(4-chlorophenyl)-2-(ethylamino)propan-1-one	CEC	*4′*	*para*	2016
3-CBC (**7**)	**3**′-**c**hloro-*N*-**b**utyl**c**athinone (*R*,*S*)-2-(butylamino)-1-(3-chlorophenyl)propan-1-one	CBC	*3′*	*meta*	**-**
4-CBC (**8**)	**4**′-**c**hloro-*N*-**b**utyl**c**athinone (*R*,*S*)-2-(butylamino)-1-(4-chlorophenyl)propan-1-one	CBC	*4′*	*para*	2017
2-Cl-PPP (**9**)	**2**′-**c**h**l**oro-α-**p**yrrolidino**p**ropio**p**henone (*R*,*S*)-1-(2-chlorophenyl)-2-(pyrrolidine-1-yl)propan-1-one	Cl-PPP	*2′*	*ortho*	**-**
3-Cl-PPP (**10**)	**3**′-**c**h**l**oro-α-**p**yrrolidino**p**ropio**p**henone (*R*,*S*)-1-(3-chlorophenyl)-2-(pyrrolidine-1-yl)propan-1-one	Cl-PPP	*3′*	*meta*	**-**
4-Cl-PPP (**11**)	**4**′-**c**h**l**oro-α-**p**yrrolidino**p**ropio**p**henone (*R*,*S*)-1-(4-chlorophenyl)-2-(pyrrolidine-1-yl)propan-1-one	Cl-PPP	*4′*	*para*	2014
2-CDC (**12**)	**2**′-**c**hloro-*N*,*N*-**d**imethyl**c**athinone (*R*,*S*)-1-(2-chlorophenyl)-2-(dimethylamino)propan-1-one	CDC	*2′*	*ortho*	**-**
3-CDC (**13**)	**3**′-**c**hloro-*N*,*N*-**d**imethyl**c**athinone (*R*,*S*)-1-(3-chlorophenyl)-2-(dimethylamino)propan-1-one	CDC	*3′*	*meta*	**-**
4-CDC (**14**)	**4**′-**c**hloro-*N*,*N*-**d**imethyl**c**athinone (*R*,*S*)-1-(4-chlorophenyl)-2-(dimethylamino)propan-1-one	CDC	*4′*	*para*	2015
3-Cl-DEC (**15**)	**3**′-**c**hloro-*N*,*N*-**d**iethyl**c**athinone (*R*,*S*)-1-(3-chlorophenyl)-2-(diethylamino)propan-1-one	Cl-DEC	*3′*	*meta*	**-**
4-Cl-DEC (**16**)	**4**′-**c**hloro-*N*,*N*-**d**iethyl**c**athinone (*R*,*S*)-1-(4-chlorophenyl)-2-(diethylamino)propan-1-one	Cl-DEC	*4′*	*para*	**-**
3-CIC (**17**)	**3**′-**c**hloro-**i**sopropyl**c**athinone (*R*,*S*)-1-(3-chlorophenyl)-2-(isopropylamino)propan-1-one	CIC	*3′*	*meta*	**-**
4-CIC (**18**)	**4**′-**c**hloro-**i**sopropyl**c**athinone (*R*,*S*)-1-(4-chlorophenyl)-2-(isopropylamino)propan-1-one	CIC	*4′*	*para*	2016
3-Cl-TBC (**19**)	**3**′-**c**hloro-***t****ert*-**b**utyl**c**athinone (*R*,*S*)-2-(*tert*-butylamino)-1-(3-chlorophenyl)propan-1-one	Cl-TBC	*3′*	*meta*	2014
4-Cl-TBC (**20**)	**4**′-**c**hloro-***t****ert*-**b**utyl**c**athinone (*R*,*S*)-2-(*tert*-butylamino)-1-(4-chlorophenyl)propan-1-one	Cl-TBC	*4′*	*para*	**-**

**Table 2:** Parameters of the dose–response curves obtained for the compounds (0.05–5 mM; 24 h) on the SH-SY5Y cell line evaluated by the MTT reduction assay are presented on the left. On the right are the results of the statistical analysis, in which cathinones from the A column that have an LC_50_ value higher than B appear in green and cathinones from the A column that have an LC_50_ value lower than B appear in red. NS represents the statistical correlations that are not significant.

Cathinones	LC_50_ (m)	[CI] (M)	Hillslope	LOAEL (mM)		B	2-CMC (1)	3-CMC (2)	4-CMC (3)	2-CEC (4)	3-CEC (5)	4-CEC (6)	3-CBC (7)	4-CBC (8)	2-Cl-PPP (9)	3-Cl-PPP (10)	4-Cl-PPP (11)	2-CDC (12)	3-CDC (13)	4-CDC (14)	3-Cl-DEC (15)	4-Cl-DEC (16)	3-CIC (17)	4-CIC (18)	3-Cl-TBC (19)	4-Cl-TBC (20)	Mephedrone (M)
					A																						
2-CMC (**1**)	1.7	[1.2–2.5]	4.3	1.0	(**1**)																						
3-CMC (**2**)	2.3	[1.8–2.9]	2.5	1.0	(**2**)		>																				
4-CMC (**3**)	1.3	[0.9–1.9]	1.6	0.1	(**3**)		<	<																			
2-CEC (**4**)	1.6	[1.3–2.0]	4.4	0.5	(**4**)		NS	<	>																		
3-CEC (**5**)	1.3	[0.8–2.3]	1.7	0.5	(**5**)		<	<	NS	<																	
4-CEC (**6**)	1.3	[1.0–1.7]	3.4	0.1	(**6**)		<	<	NS	<	NS																
3-CBC (**7**)	0.7	[0.5–1.0]	1.4	0.1	(**7**)		<	<	<	<	<	<															
4-CBC (**8**)	0.6	[0.4–1.0]	10.5	0.5	(**8**)		<	<	<	<	<	<	<														
2-Cl-PPP (**9**)	1.6	[1.2–1.9]	2.4	0.5	(**9**)		NS	<	>	NS	>	>	>	>													
3-Cl-PPP (**10**)	2.5	[2.0–2.9]	1.7	0.1	(**10**)		>	NS	>	>	>	>	>	>	>												
4-Cl-PPP (**11**)	1.8	[1.2–2.5]	0.9	0.5	(**11**)		NS	<	>	NS	>	>	>	>	>	<											
2-CDC (**12**)	1.3	[1.1–1.5]	12.4	1.0	(**12**)		<	<	NS	<	NS	NS	>	>	<	<	<										
3-CDC (**13**)	1.8	[1.2–2.7]	6.3	1.0	(**13**)		NS	<	>	>	>	>	>	>	>	<	NS	>									
4-CDC (**14**)	1.5	[1.1–2.1]	5.6	1.0	(**14**)		NS	<	>	NS	>	>	>	>	NS	<	<	>	<								
3-Cl-DEC (**15**)	1.2	[1.0–1.5]	4.3	1.0	(**15**)		<	<	NS	<	NS	NS	>	>	<	<	<	NS	<	<							
4-Cl-DEC (**16**)	1.0	[1.0–1.1]	17.4	1.0	(**16**)		<	<	<	<	<	<	>	>	<	<	<	<	<	<	<						
3-CIC (**17**)	1.6	[1.2–2.1]	4.4	1.0	(**17**)		NS	<	>	NS	>	>	>	>	NS	<	NS	>	<	NS	>	>					
4-CIC (**18**)	1.4	[1.0–2.0]	4.5	1.0	(**18**)		<	<	NS	<	NS	NS	>	>	NS	<	<	NS	<	NS	>	>	<				
3-Cl-TBC (**19**)	1.0	[0.9–1.2]	3.5	0.5	(**19**)		<	<	<	<	<	<	>	>	<	<	<	<	<	<	<	NS	<	<			
4-Cl-TBC (**20**)	1.3	[1.1–1.7]	2.4	0.5	(**20**)		<	<	NS	<	NS	NS	>	>	<	<	<	NS	<	<	NS	>	<	NS	>		
Mephedrone (**M)**	2.1	[1.4–3.1]	2.3	0.5	(**M**)		>	NS	>	>	>	>	>	>	>	<	>	>	>	>	>	>	>	>	>	>	

**Table 3:** Parameters of dose–response curves obtained for cathinones (0.03–2 mM) on acetylcholinesterase inhibition, evaluated by the Ellman assay. On the right are the results of the statistical analysis, in which cathinones from the A column that have an IC_50_ value higher than B appear in green and cathinones from the A column that have an IC_50_ value lower than B appear in red. NS represents the statistical correlations that are not significant; ND represents the statistical correlations that were not possible to determine; * 2 mM showed 47% inhibition; ** 2 mM showed 48% inhibition.

Cathinones	IC_50_ (m)	[CI] (M)	Hillslope	LOAEL (mM)		B	2-CMC (1)	3-CMC (2)	4-CMC (3)	2-CEC (4)	3-CEC (5)	4-CEC (6)	3-CBC (7)	4-CBC (8)	2-Cl-PPP (9)	3-Cl-PPP (10)	4-Cl-PPP (11)	2-CDC (12)	3-CDC (13)	4-CDC (14)	3-Cl-DEC (15)	4-Cl-DEC (16)	3-CIC (17)	4-CIC (18)	3-Cl-TBC (19)	4-Cl-TBC (20)	Mephedrone (M)
					A																						
2-CMC (**1**)	0.7	[0.4–1.3]	2.5	0.250	(**1**)																						
3-CMC (**2**)	0.8	[0.6–0.9]	3.7	0.500	(**2**)		>NS																				
4-CMC (**3**)	~	2 *	1.5	0.250	(**3**)		>	>																			
2-CEC (**4**)	0.6	[0.5–0.6]	4.3	0.500	(**4**)		NS	<	<																		
3-CEC (**5**)	0.8	[0.5–1.2]	1.3	0.250	(**5**)		NS	NS	<	>																	
4-CEC (**6**)	1.2	[1.0–1.4]	1.2	0.250	(**6**)		>	>	<	>	>																
3-CBC (**7**)	0.4	[0.3–0.5]	1.4	0.125	(**7**)		<	<	<	<	<	<															
4-CBC (**8**)	1.3	[1.0–1.8]	5.1	0.100	(**8**)		>	>	<	>	>	NS	>														
2-Cl-PPP (**9**)	0.3	[0.2–0.5]	1.5	0.125	(**9**)		<	<	<	<	<	<	<	<													
3-Cl-PPP (**10**)	0.4	[0.2–0.8]	1.8	0.125	(**10**)		<	<	<	<	<	<	NS	<	>												
4-Cl-PPP (**11**)	0.5	[0.3–0.7]	1.1	0.250	(**11**)		<	<	<	<	<	<	NS	<	>	NS											
2-CDC (**12**)	0.7	[0.6–0.8]	2.5	0.250	(**12**)		NS	NS	<	NS	NS	<	>	<	>	>	>										
3-CDC (**13**)	0.9	[0.9–1.1]	1.2	0.250	(**13**)		>	>	<	>	>	>	>	<	>	>	>	>									
4-CDC (**14**)	0.7	[0.7–0.8]	2.9	0.50	(**14**)		NS	NS	<	>	NS	<	>	<	>	>	>	>NS	>								
3-Cl-DEC (**15**)	0.3	[0.1–0.6]	0.9	0.063	(**15**)		<	<	<	<	<	<	<	<	NS	<	<	<	<	<							
4-Cl-DEC (**16**)	0.1	[0.1–0.2]	0.8	0.063	(**16**)		<	<	<	<	<	<	<	<	<	<	<	<	<	<	<						
3-CIC (**17**)	0.2	[0.1–0.2]	0.8	0.063	(**17**)		<	<	<	<	<	<	<	<	<	<	<	<	<	<	<	NS					
4-CIC (**18**)	1.1	[0.7–1.7]	2.0	0.375	(**18**)		>	>	<	>	>	NS	>	NS	>	>	>	>	NS	>	>	>	>				
3-Cl-TBC (**19**)	1.0	[0.9–1.2]	0.9	0.250	(**19**)		>	>	<	>	>	NS	>	<	>	>	>	>	NS	>	>	>	>	NS			
4-Cl-TBC (**20**)	~	2 **	2.7	0.500	(**20**)		>	>	ND	>	>	>	>	>	>	>	>	>	>	>	>	>	>	>	>		
Mephedrone (**M)**	0.8	[0.8–0.9]	8.6	0.750	(**M**)		NS	NS	<	>	NS	<	>	<	>	>	>	NS	NS	NS	>	>	<	<	<	<	

## Data Availability

Data is available from the corresponding author upon request.

## Supplementary Materials

The following supporting information can be downloaded at https://www.mdpi.com/article/10.3390/ijms26083540/s1.
