# Supplementary material for: Synthesis, Characterization, and Biological Effects of Chloro-Cathinones: Toxicity and Potential Neurological Impact
Source: Int J Mol Sci. 2025 Apr 9;26(8):3540. doi: 10.3390/ijms26083540 (PMC12027149; doi:10.3390/ijms26083540)
Supplement: Supplementary file 1 [file ijms-26-03540-s001.zip › ijms-3545894-supplementary April10 Updated-highlight.pdf]

Supplementary Materials

Synthesis, Characterization, and Biological Effects of Chloro-Cathinones: Toxicity and Potential Neurological Impact

Contents

1. **NMR Data**.. **S2**
   Table S1- NMR data for CMC (**1**-**3**), DMSO-d_6.
   Table S2- NMR data for CEC (**4**-**6**), DMSO-d_6.
   Table S3- NMR data for CBC (**7**-**8**), DMSO-d_6.
   Table S4- NMR data for Cl-PPP (**9**-**11**), DMSO-d_6.
   Table S5- NMR data for CDC (**12**-**14**), DMSO-d_6.
   Table S6- NMR data for Cl-DEC (**15**-**16**), DMSO-d_6.
   Table S7- NMR data for CIC (**17**-**18**), DMSO-d_6.
   Table S8- NMR data for Cl-TBC (**19**-**20**), DMSO-d_6.
2. **HR-ESI-MS Data**.. **S6**
   Table S9 - HRESI-MS for of synthesized cathinones (**1**-**20**).
3. **GC-EI-MS Data**.. **S7**
   Figure S1- GC-EI-MS data for CMC (**1**-**3**).
   Figure S2- GC-EI-MS data for CEC (**4**-**6**).
   Figure S3- GC-EI-MS data for CBC (**7**-**8**).
   Figure S4- GC-EI-MS data for Cl-PPP (**9**-**11**).
   Figure S5- GC-EI-MS data for CDC (**12**-**14**).
   Figure S6- GC-EI-MS data for Cl-DEC (**15**-**16**).
   Figure S7- GC-EI-MS data for CIC (**17**-**18**).
   Figure S8- GC-EI-MS data for Cl-TBC (**19**-**20**).
4. **Molecular docking studies**.. **S15**
   Table S10- Molecular docking results of 3-Cl-DEC (**15**).
   Table S11- Molecular docking results of 4-Cl-DEC (**16**).
   Table S12- Molecular docking results of 3-CIC (**17**).
   Table S13- Molecular docking results of 4-CIC (**18**).

1. NMR Data

Table S1- NMR data for CMC (**1-3**), DMSO-*d*6.

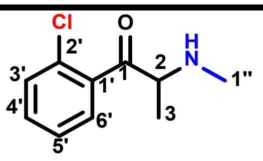

2-CMC (**1**)

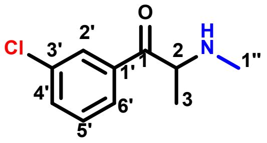

3-CMC (**2**)

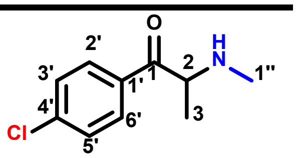

4-CMC (**3**)

Position	δC	δH, m, *J* (Hz)	δC	δH, m, *J* (Hz)	δC	δH, m, *J* (Hz)
**1**	197.41	-	195.56	-	195.53	-
**2**	60.50	5.00; m	58.33	5.21; m	58.27	5.17; m
**3**	13.42	1.36; d; 7.3	15.18	1.44; d; 7.0	15.27	1.44; d; 7.0
**1′**	134.19	-	134.89	-	131.71	-
**2′**	130.85	-	128.43	8.07; s	130.79	8.06; d; 8.2
**3′**	131.02	7.64; m	134.16	-	129.39	7.69; d; 8.2
**4′**	133.78	7.64; m	134.40	7.82; d; 7.6	139.68	-
**5′**	127.64	7.54; m	131.28	7.64; t; 7.9	129.39	7.69; d; 8.2
**6′**	129.97	7.92; d; 7.7	127.54	8.00; d; 8.0	130.79	8.06; d; 8.2
**1′′**	30.54	2.63; s	30.65	2.59; s	30.68	2.59; m
**NH**	-	9.40/9.79; brs	-	9.51; brs	-	9.45; brs

Table S2- NMR data for CEC (**4-6**), DMSO-*d*6.

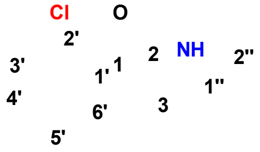

2-CEC (**4**)

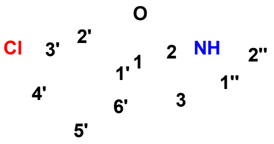

3-CEC (**5**)

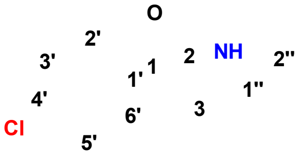

4-CEC (**6**)

Position	δC	δH, m, *J* (Hz)	δC	δH, m, *J* (Hz)	δC	δH, m, *J* (Hz)
**1**	197.45	-	195.54	-	195.50	-
**2**	59.16	5.01; m	56.78	5.25; m	56.74	5.22; m
**3**	13.76	1.37; d; 7.3	15.44	1.45; d; 7.1	15.52	1.45; d; 7.1
**1′**	134.10	-	134.85	-	131.67	-
**2′**	130.90	-	128.46	8.11; brs	130.83	8.11; brs
**3′**	131.06	7.65; m	134.15	-	129.39	7.83; d; 8.0
**4′**	133.83	7.65; m	134.42	7.83; d; 8.0	139.72	-
**5′**	127.64	7.55; m	131.24	7.65; t; 7.9	129.39	7.83; d; 8.0
**6′**	129.97	7.91; d; 7.7	127.55	8.03; d; 7.9	130.83	8.11; brs
**1′′**	40.31	2.99/3.09; brs	40.28	2.92/3.04; brs	40.31	2.92/3.04; brs
**2′′**	11.23	1.29; t; 7.2	11.19	1.27; t; 7.2	11.20	1.27; t; 7.2
**NH**	-	9.24/9.58; brs	-	9.21/9.70; brs	-	9.21/9.70; brs

Table S3- NMR data for CBC (**7**-**8**), DMSO-*d*6.

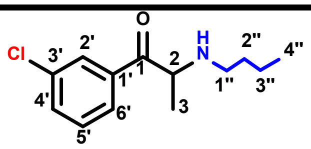

3-CBC (**7**)

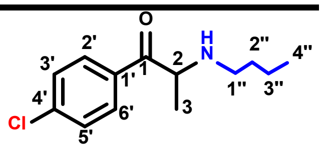

4-CBC (**8**)

**Position**	δ_C	δ_H, **m,** ***J*** **(Hz)**	δ_C	δ_H, **m,** ***J*** **(Hz)**
**1**	195.59	-	195.50	-
**2**	57.39	5.25; m	57.30	5.23; q; 7.0
**3**	15.57	1.45; d; 7.2	15.61	1.43; d; 7.1
**1′**	134.85	-	131.64	-
**2′**	128.52	8.10; s	130.87	8.08; d; 8.6
**3′**	134.56	-	129.44	7.70; d; 8.6
**4′**	134.25	7.83; d; 8.0	139.81	-
**5′**	131.36	7.65; t; 7.9	129.44	7.70; d; 8.6
**6′**	127.63	8.01; d; 7.9	130.87	8.08; d; 8.6
**1″**	45.06	2.92; m	45.02	2.93; m
**2″**	27.72	1.66; m	27.70	1.65; m
**3″**	19.41	1.34; m	19.38	1.35; m
**4″**	13.60	0.90; t; 7.3	13.57	0.90; t; 7.4
**NH**	-	9.42/ 9.02; brs	-	9.29/ 8.96; brs

Table S4- NMR data for Cl-PPP (**9**-**11**), DMSO-*d*6.

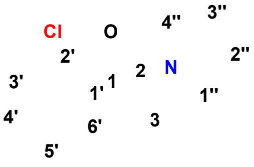

2-Cl-PPP (**9**)

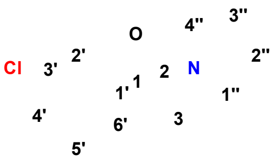

3-Cl-PPP (**10**)

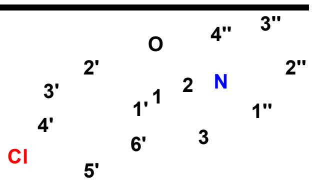

4-Cl-PPP (**11**)

**Position**	δ_C	δ_H, **m,** ***J*** **(Hz)**	δ_C	δ_H, **m,** ***J*** **(Hz)**	δ_C	δ_H, **m,** ***J*** **(Hz)**
**1**	197.43	-	195.52	-	195.46	-
**2**	66.47	5.33; m	64.30	5.61; m	64.17	5.52; m
**3**	14.15	1.42; d; 7.2	15.75	1.49; d; 7.1	15.77	1.49; d; 7.1
**1′**	134.00	-	134.81	-	131.65	-
**2′**	131.07	-	128.59	8.09; brs	130.86	8.06; d; 8.6
**3′**	131.20	7.67; m	134.12	-	129.40	7.72; d; 8.6
**4′**	134.11	7.67; m	134.57	7.85; brd; 7.6	139.90	-
**5′**	127.69	7.57; m	131.26	7.67; t; 7.9	129.40	7.72; d; 8.6
**6′**	130.11	7.97; d; 7.6	127.59	8.01; d; 7.2	130.86	8.06; d; 8.6
**1″/4″**	51.78<br>53.24	3.23/3.60; m	51.88<br>53.28	3.23/3.62; m<br>3.12/3.53; m	51.82<br>53.22	3.21/3.62; m<br>3.10/3.53; m
**2″/3″**	23.06<br>23.22	1.95/2.04; m	23.04<br>23.16	1.94; m	23.02<br>23.15	1.95; m
**NH**	-	10.52; brs	-	10.45; brs	-	10.49; brs

Table S5- NMR data for CDC (**12-14**), DMSO-d_6.

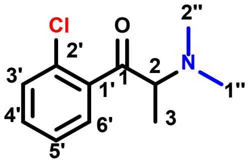

2-CDC (**12**)

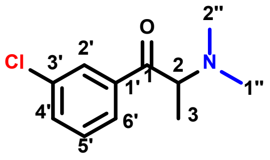

3-CDC (**13**)

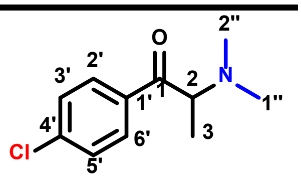

4-CDC (**14**)

**Position**	**δC**	**δH, m, *J* (Hz)**	**δC**	**δH, m, *J* (Hz)**	**δC**	**δH, m, *J* (Hz)**
**1**	197.17	-	195.40	-	195.46	-
**2**	66.10	5.27; q; 7.4	63.99	5.45; q; 7.2	64.06	5.37; m
**3**	11.57	1.41; d; 7.4	13.47	1.48; d; 7.2	13.42	1.47; d; 7.1
**1′**	134.63	-	135.35	-	132.13	-
**2′**	130.66	-	128.51	8.08; brs	130.82	8.05; d; 8.5
**3′**	130.93	7.64; m	134.13	-	129.37	7.70; d; 8.5
**4′**	133.75	7.64; m	134.47	7.84; brd; 8.0	139.79	-
**5′**	127.60	7.53; m	131.26	7.65; t; 7.9	129.37	7.70; d; 8.5
**6′**	129.76	7.93; d; 7.7	127.56	7.99; d; 7.9	130.82	8.05; d; 8.5
**1″/2″**	39.78<br>41.28	2.88; s	39.99<br>41.91	2.86; m	39.91<br>42.17	2.84; brs
**NH**	-	10.93; brs	-	10.63; brs	-	10.42; brs

Table S6- NMR data for Cl-DEC (**15-16**), DMSO-d_6.

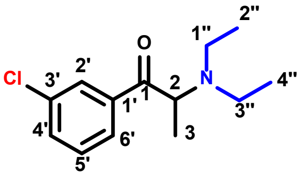

3-Cl-DEC (**15**)

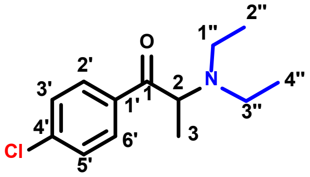

4-Cl-DEC (**16**)

**Position**	**δC**	**δH, m, *J* (Hz)**	**δC**	**δH, m, *J* (Hz)**
**1**	195.29	-	195.20	-
**2**	61.67	5.39; *brt*; 6.4	61.40	5.40; *m*
**3**	14.01	1.49; *d*; 6.4	14.02	1.50; *d*; 7.1
**1′**	134.16	-	132.02	-
**2′**	128.68	8.18; *s*	131.02	8.16; *d*; 8.7
**3′**	135.19	-	129.32	7.70; *d*; 8.7
**4′**	134.59	7.84; *brd*; 7.7	139.85	-
**5′**	131.23	7.65; *t*; 7.9	129.32	7.70; *d*; 8.7
**6′**	127.75	8.08; *brd*; 7.6	131.02	8.16; *d*; 8.7
**1″**	44.68	3.14; *m*	44.73	3.15/3.34; *m*
**2″**	8.50	1.29; *m*	8.57	1.30; *m*
**3″**	46.69	3.35/3.14; *m*	46.56	3.15/3.26; *m*
**4″**	10.08	1.29; *m*	10.05	1.30; *m*
**NH**	-	10.05; *brs*	-	10.12; *brs*

Table S7- NMR data for CIC (**17-18**), DMSO-d_6.

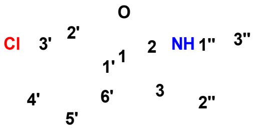

3-CIC (**17**)

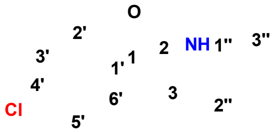

4-CIC (**18**)

**Position**	δ_C	**δ_H, m, *J* (Hz)**	δ_C	**δ_H, m, *J* (Hz)**
**1**	195.26	-	195.33	-
**2**	54.65	5.27; m	54.57	5.25; m
**3**	15.77	1.47; d; 7.1	15.89	1.47; d; 7.1
**1′**	134.16	-	131.52	-
**2′**	128.52	8.20; brs	130.97	8.17; d; 8.6
**3′**	134.68	-	129.38	7.69; d; 8.6
**4′**	134.44	7.83; d; 8.0	139.83	-
**5′**	131.17	7.65; t; 7.9	129.38	7.69; d; 8.6
**6′**	127.61	8.10; d; 8.0	130.97	8.17; d; 8.6
**1″**	48.07	3.35; m	48.05	3.36; m
**2″/3″**	18.86<br>18.95	1.31; d; 6.5	18.93<br>18.99	1.30; d; 6.4
**NH**	-	9.04/9.43; brs	-	9.03/9.45; brs

Table S8- NMR data for Cl-TBC (**19-20**), DMSO-d_6.

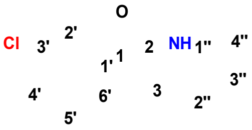

3-Cl-TBC (**19**)

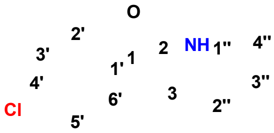

4-Cl-TBC (**20**)

**Position**	δ_C	**δ_H, m, *J* (Hz)**	δ_C	**δ_H, m, *J* (Hz)**
**1**	195.52	-	195.72	-
**2**	52.96	5.32; m	52.86	5.26; m
**3**	18.09	1.52; d; 7.0	18.25	1.49; d; 7.0
**1′**	134.08	-	130.99	-
**2′**	128.66	8.27; brs	131.12	8.23; d; 8.5
**3′**	134.34	-	129.56	7.70; d; 8.5
**4′**	134.80	7.86; d; 8.0	140.20	-
**5′**	131.34	7.66; t; 7.9	129.56	7.70; d; 8.5
**6′**	127.81	8.17; d; 7.8	131.12	8.23; d; 8.5
**1″**	58.17	-	58.08	-
**2″/3″/4″**	25.99	1.31; brs	26.06	1.29; m
**NH**	-	8.63/9.60; brs	-	8.56/9.38; m

2. HR-ESI-MS Data

Table S9 – HR-ESI-MS Data synthesized cathinones (**1**-**20**).

Cathinone	[Molecule + H]+	Exact mass	Accurate mass (Δ ppm)
2-CMC (**1**)	$C_{10}H_{13}ClNO^+$	198.0680	198.0680 (0.0)
3-CMC (**2**)	$C_{10}H_{13}ClNO^+$	198.0680	198.0682 (-1.0)
4-CMC (**3**)	$C_{10}H_{13}ClNO^+$	198.0680	198.0680 (0.0)
2-CEC (**4**)	$C_{11}H_{15}ClNO^+$	212.0837	212.0842 (-2.4)
3-CEC (**5**)	$C_{11}H_{15}ClNO^+$	212.0837	212.0842 (-2.4)
4-CEC (**6**)	$C_{11}H_{15}ClNO^+$	212.0837	212.0839 (-0.9)
3-CBC (**7**)	$C_{13}H_{19}ClNO^+$	240.1150	240.1149 (-0.3)
4-CBC (**8**)	$C_{13}H_{19}ClNO^+$	240.1150	240.1149 (+0.4)
2-Cl-PPP (**9**)	$C_{13}H_{17}ClNO^+$	238.0993	238.0997 (-1.7)
3-Cl-PPP (**10**)	$C_{13}H_{17}ClNO^+$	238.0993	238.0996 (-1.3)
4-Cl-PPP (**11**)	$C_{13}H_{17}ClNO^+$	238.0993	238.1000 (-2.9)
2-CDC (**12**)	$C_{11}H_{15}ClNO^+$	212.0837	212.0835 (+0.9)
3-CDC (**13**)	$C_{11}H_{15}ClNO^+$	212.0837	212.0840 (-1.4)
4-CDC (**14**)	$C_{11}H_{15}ClNO^+$	212.0837	212.0839 (-0.9)
3-Cl-DEC (**15**)	$C_{13}H_{19}ClNO^+$	240.1150	240.1149 (+0.4)
4-Cl-DEC (**16**)	$C_{13}H_{19}ClNO^+$	240.1150	242.1149 (0.0)
3-CIC (**17**)	$C_{12}H_{17}ClNO^+$	226.0993	226.0996 (-1.3)
4-CIC (**18**)	$C_{12}H_{17}ClNO^+$	226.0993	226.0994 (-0.4)
3-Cl-TBC (**19**)	$C_{13}H_{19}ClNO^+$	240.1150	240.1154 (-1.7)
4-Cl-TBC (**20**)	$C_{13}H_{19}ClNO^+$	240.1150	240.1152 (-0.8)

3. GC-ESI-MS Data

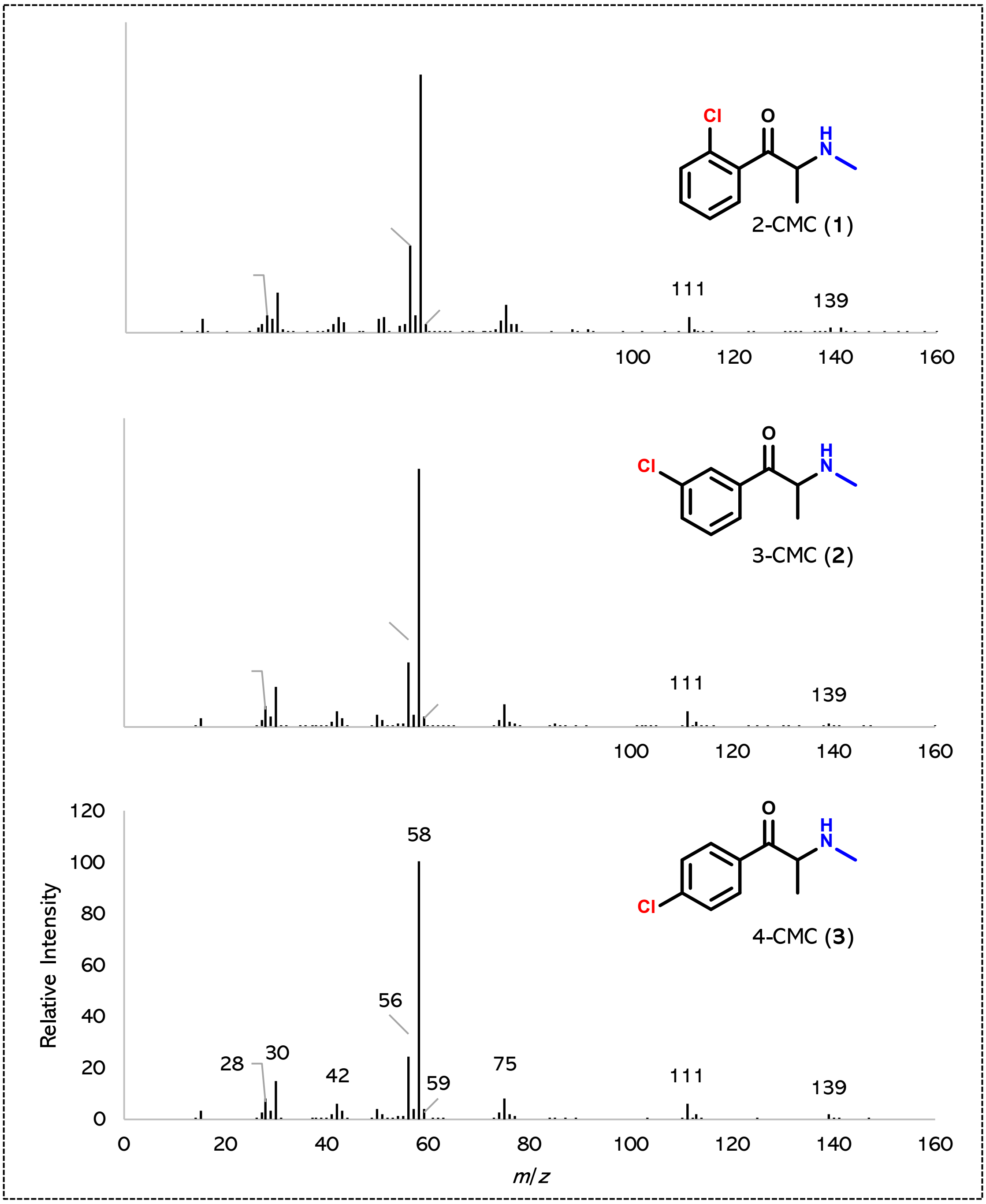

Figure S1- GC-EI-MS data for CMC (**1**-**3**).

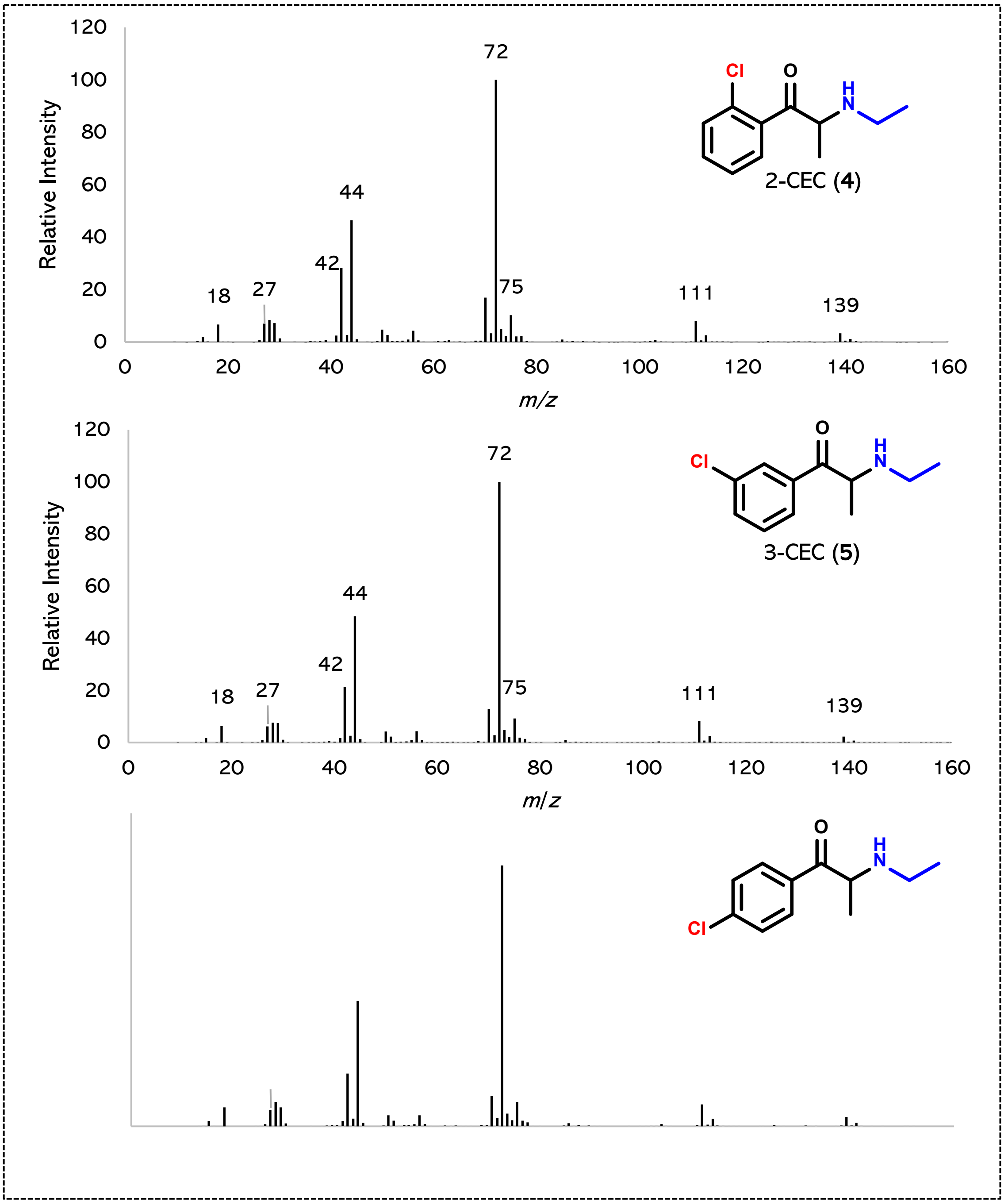

Figure S2- GC-EI-MS data for CEC (**4**-**6**).

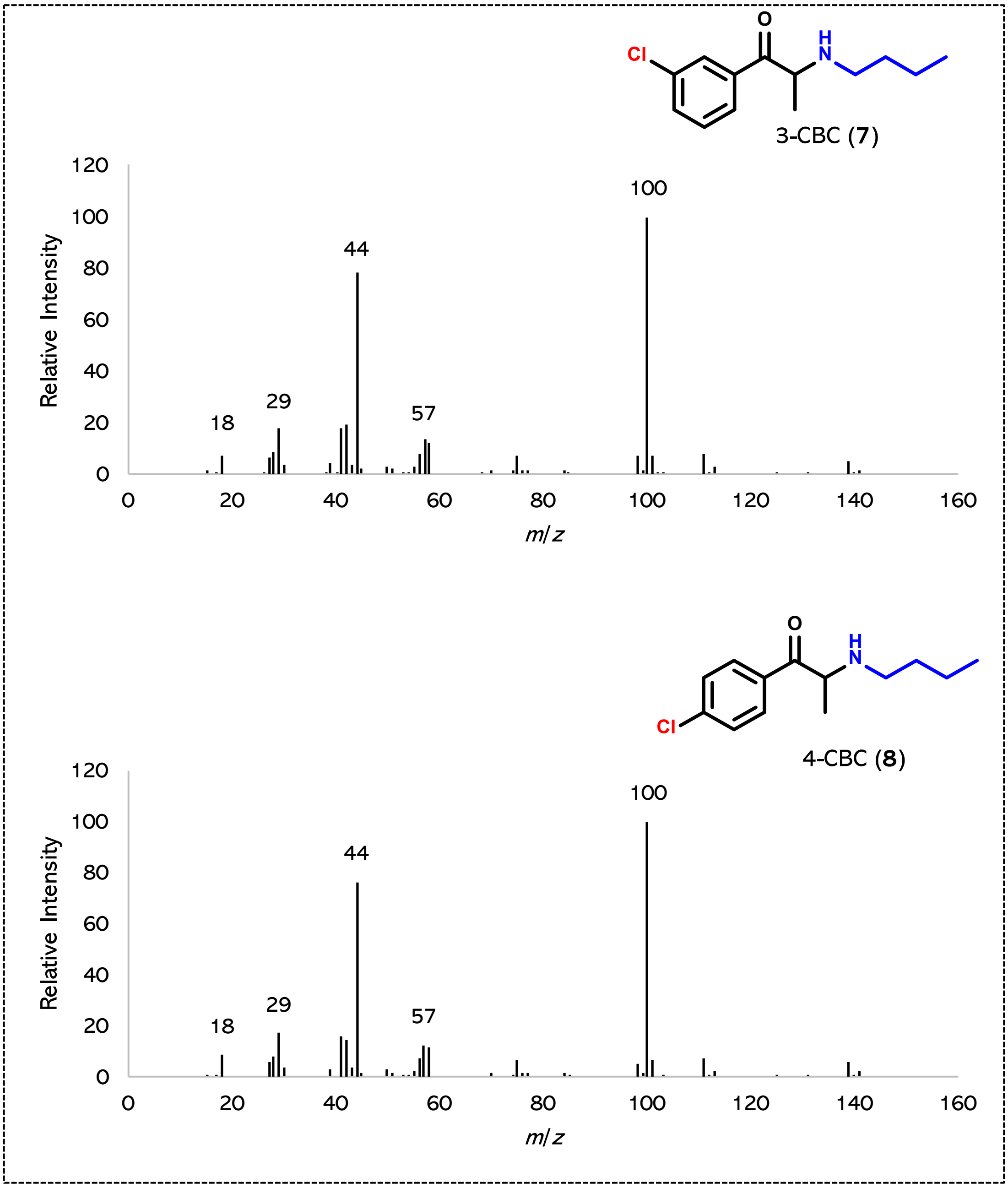

Figure S3- GC-EI-MS data for CBC (**7**-**8**).

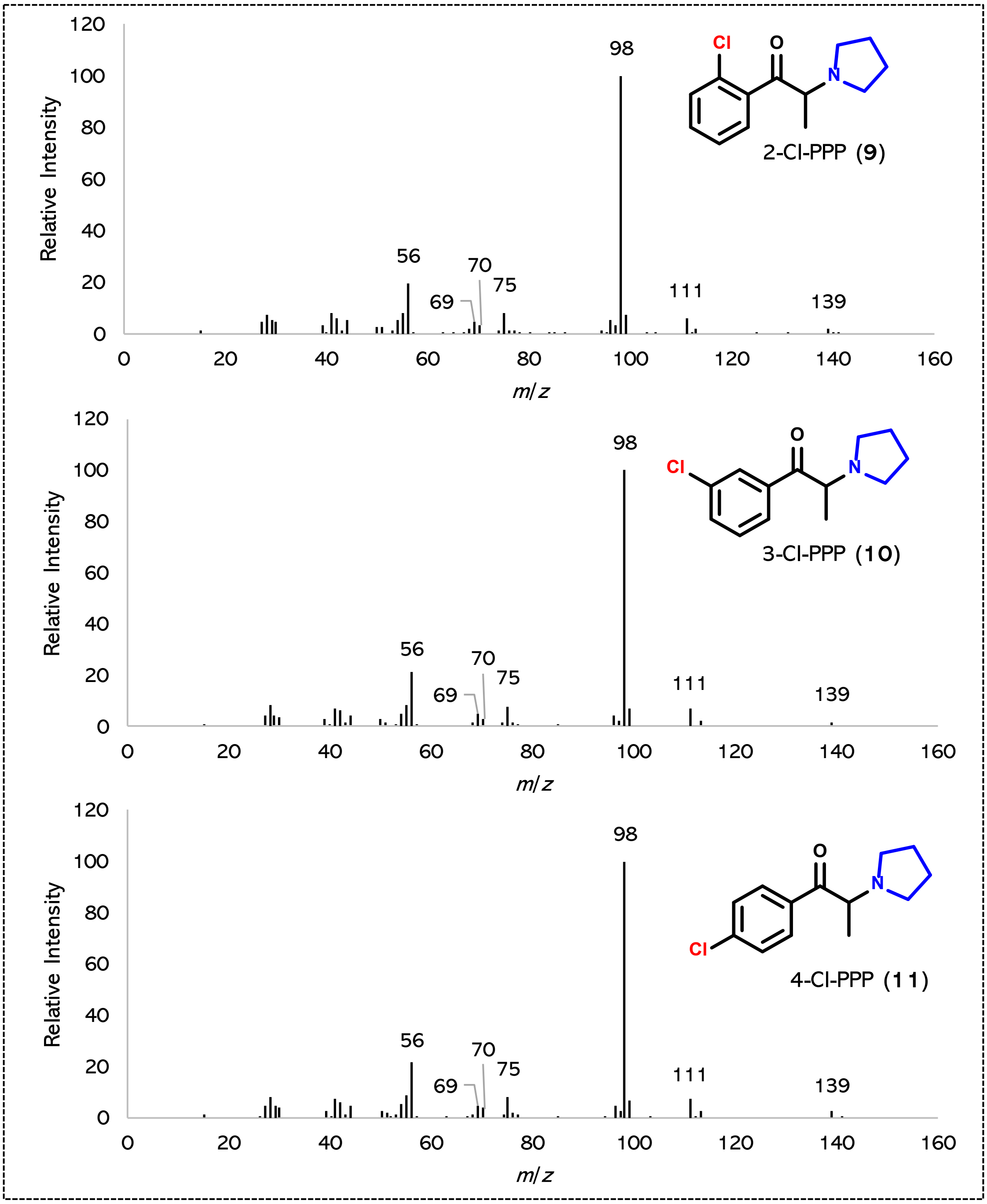

Figure S4- GC-EI-MS data for Cl-PPP (**9**-**11**).

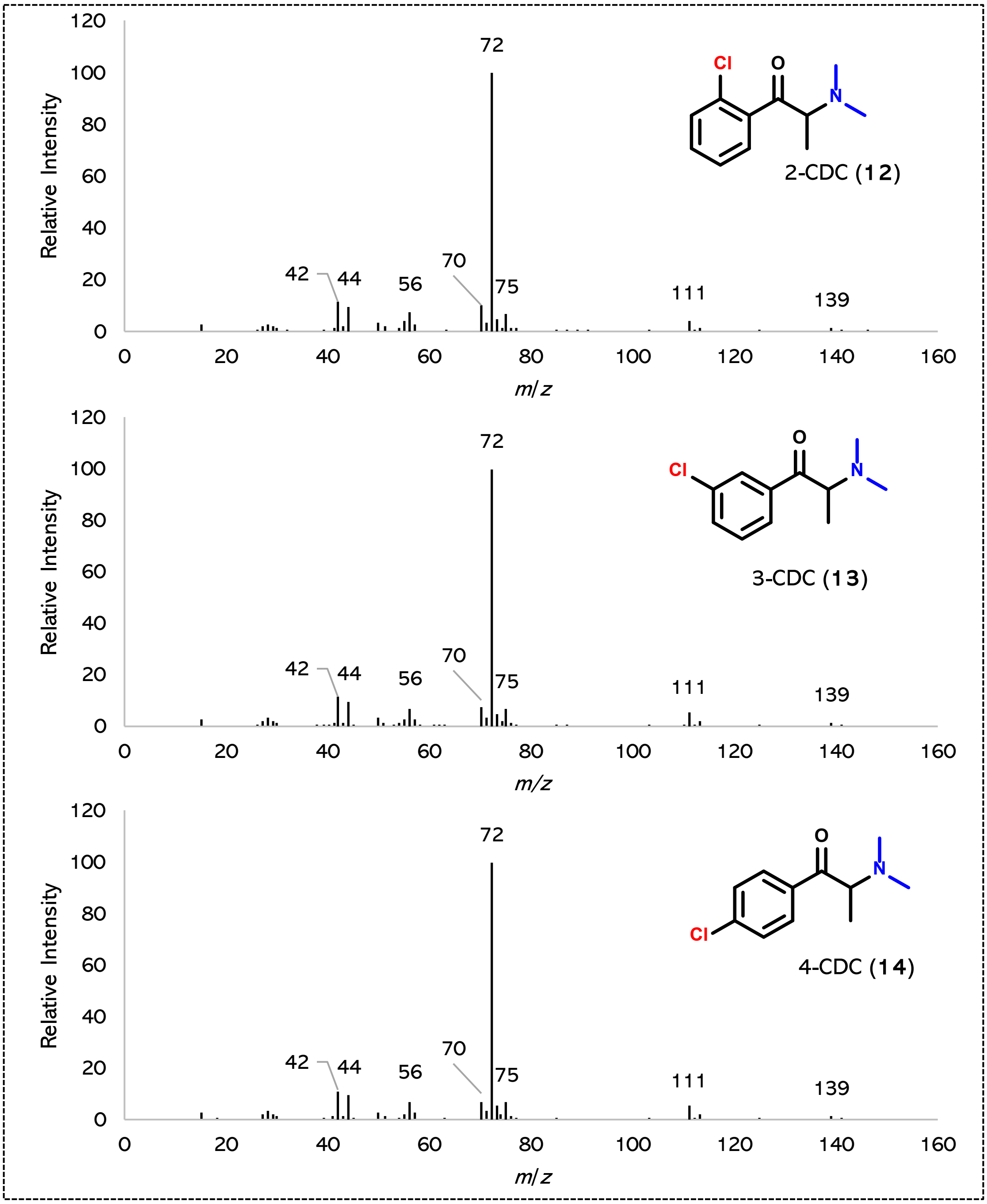

Figure S5- GC-EI-MS data for CDC (**12**-**14**).

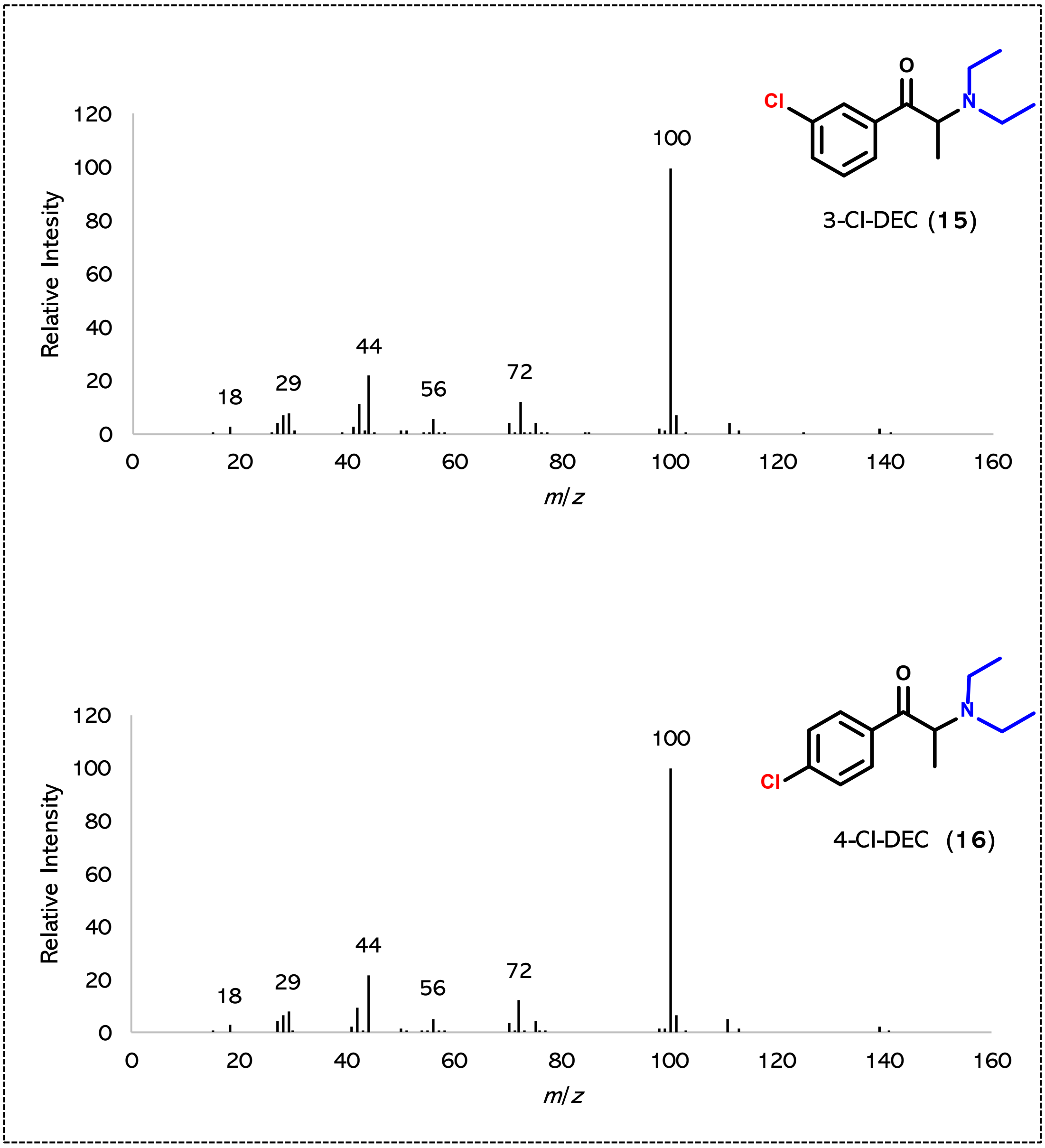

Figure S6- GC-EI-MS data for Cl-DEC (**15**-**16**).

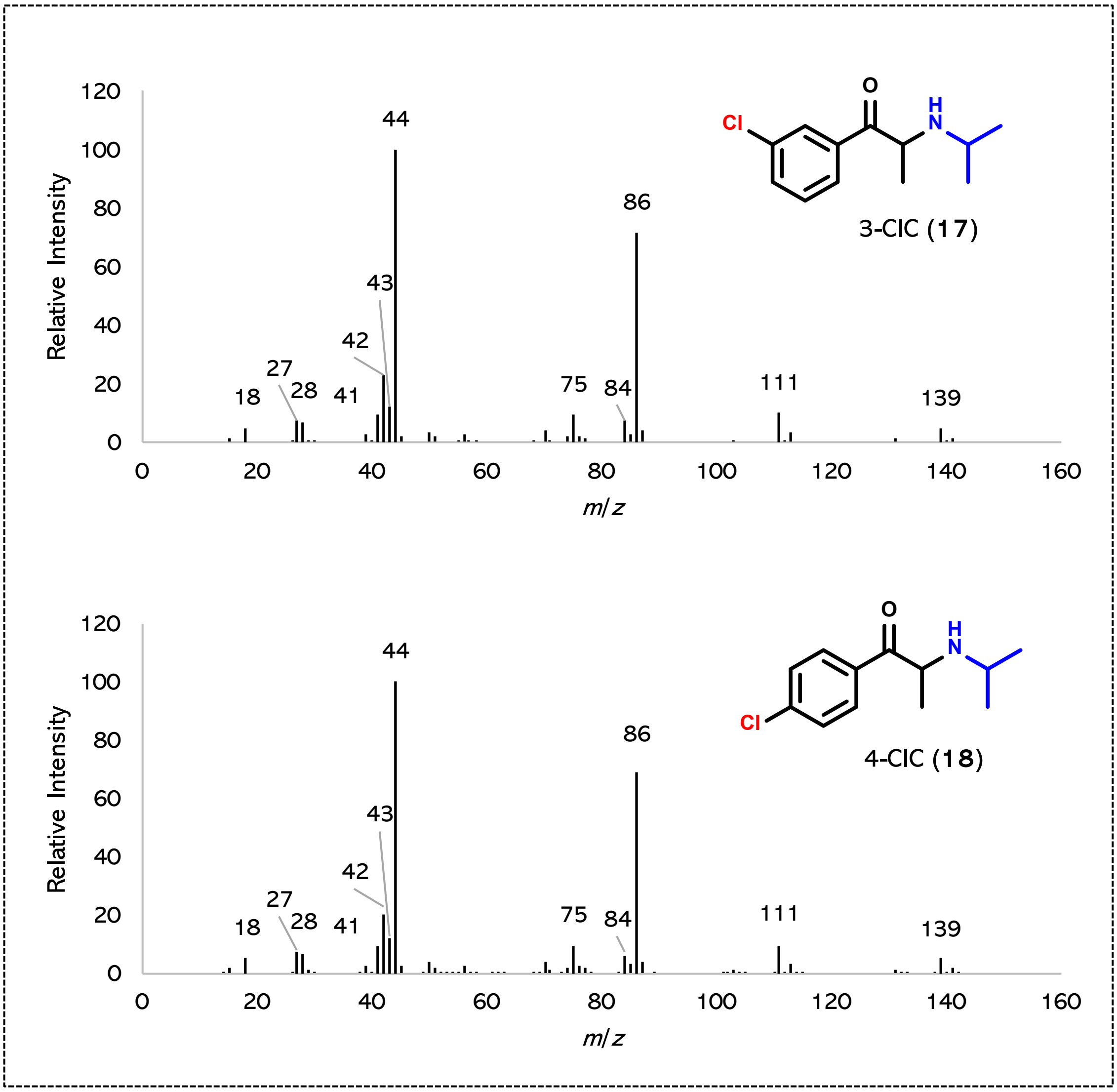

Figure S7- GC-EI-MS data for ClC (**17**-**18**).

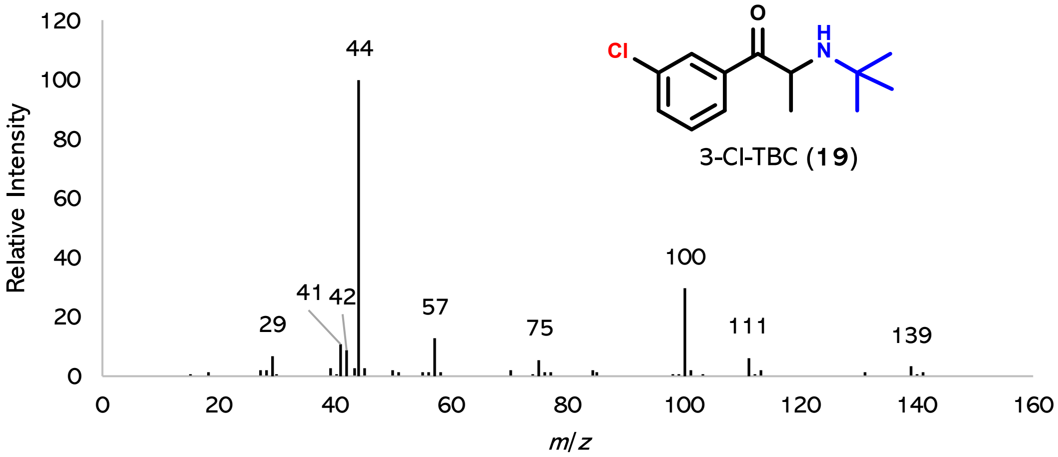

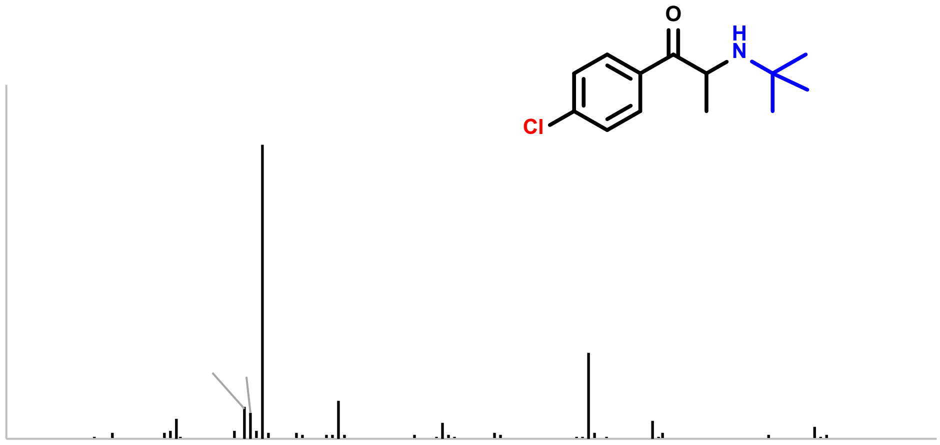

Figure S8- GC-EI-MS data for Cl-TBC (**19**-**20**).

4. Molecular Docking Studies

Table S10- Molecular docking results of 3-Cl-DEC (**15**)

AA residue	Cathinone Atom/group	Interaction type	Distance(Å)
Tyr328	O=C	Hydrogen bond	2.17
Trp83	NH^+	π-cation	4.61
Trp83	$NH^+(CH_2\underline{CH_3})_2$	π-sigma	3.87/3.84
Tyr332	Cl	π-alkyl	3.94
Tyr332	Ar	π-π stacking	3.89
Trp277	Cl	π-alkyl	5.14
Tyr121	Cl	π-alkyl	5.34

Table S11- Molecular docking results of 4-Cl-DEC (**16**)

AA residue	Cathinone Atom/group	Interaction type	Distance(Å)
Trp83	NH^+	π-cation	4.88
Trp83	$NH^+(CH_2\underline{CH_3})_2$	π-sigma	4.49
Trp83	O=C$\underline{CH}$CH3	π-sigma	4.51
Tyr328	NH^+	π-sigma	4.78
Tyr328	O=C	H bond	2.11
Tyr332	Cl	π-alkyl	3.96
Tyr332	Ar	π-π stacking	3.88
Phe329	Cl	π-alkyl	4.89

Table S12- Molecular docking results of 3-CIC (**17**)

AA residue	Cathinone Atom/group	Interaction type	Distance(Å)
Trp83	NH_2^+ $CH(\underline{CH_3})_2$	π-sigma	3.97
Tyr328	O=C	Hydrogen bond	2.16
Trp277	Cl	π-alkyl	5.36
Tyr332	Ar	π-π stacking	3.89
Tyr332	Cl	π-alkyl	3.92
Tyr121	Cl	π-alkyl	5.49
Asp71	NH_2^+	Electrostatic interaction	5.30

Table S13- Molecular docking results of 4-CIC (**18**)

AA residue	Cathinone Atom/group	Interaction type	Distance (Å)
Arg287	NH_2^+	Hydrogen bond	2.27
Trp277	NH_2^+ $CH(\underline{CH_3})_2$	π-sigma	3.91
Tyr332	Ar	π-π stacking	4.22
Tyr332	Cl	π-alkyl	3.93
Tyr332	NH_2^+	Hydrogen bond	2.19
Phe329	Cl	π-alkyl	4.92
Phe288	Ar	π-π stacking	5.91
